# Deep learning–enabled versatile shape perception for soft robots via single-ended multimode fiber

**DOI:** 10.1126/sciadv.aef6263

**Published:** 2026-06-12

**Authors:** Zhaofan He, Lele Wang, Haidi Geng, Zhengyang Lu, Tiantian He, Hongkun Zhong, Hailong Zhang, Runfeng Zhu, Qingxiang Zhao, Yuan Meng, Dan Li, Ping Yan, Qiang Liu, Qirong Xiao

**Affiliations:** ^1^ Department of Precision Instrument, Tsinghua University, Beijing 100084, China.; ^2^ State Key Laboratory of Precision Space-time Information Sensing Technology, Beijing 100084, China.; ^3^ Weixian College, Tsinghua University, Beijing 100084, China.; ^4^ West China Biomedical Big Data Center, West China Hospital, Sichuan University, Chengdu 610041, China.

## Abstract

The evolution of soft robots into embodied intelligent systems relies fundamentally on precise proprioception. However, a universal solution for capturing continuous deformations during diverse interactions, particularly in spatially confined interventional scenarios, remains lacking. Here, we introduce a deep learning–enabled versatile shape perception method based on a single-ended multimode fiber (MMF). By leveraging the intrinsic integration advantages of optics, our minimalist reflective architecture physically eliminates the dependence on complex demodulation units and distal devices. Furthermore, treating chaotic optical speckle fields as data streams encoding high-dimensional shape information, reconfigurable neural decoders resolve a single physical channel into versatile perception modes tailored to heterogeneous tasks: discrete state confirmation on soft grippers (>99% accuracy), continuous shape tracking on bionic dexterous hands (~5-fold spatial resolution enhancement), and intuitive 3D morphological reconstruction of soft surgical robots (IoU>0.93). Overall, our work establishes a versatile framework for breaking hardware adaptability limits via computation, laying a solid foundation for closed-loop control in digital twins of soft robots.

## Teaser

Decoding chaotic speckles from minimalist fiber hardware empowers diverse soft robots with precise proprioception.

## INTRODUCTION

As robotic systems evolve toward embodied intelligence (*1*, *2*), the boundary between perception and action is blurring. Soft robots, characterized by continuous media and hyper-redundant degrees of freedom, offer unparalleled adaptability in unstructured environments (*3*, *4*). However, the same inherent flexibility that enables safe interaction also makes their motion states notoriously hard to predict during external interactions (*5*, *6*). Crucially, whether stemming from external loads, contact pressures, or internal actuation, these physical processes manifest primarily and most directly as geometric deformations of the continuum. Shape serves not only as the spatiotemporal carrier of a soft robot’s state but also as the fundamental basis for deducing other physical quantities such as force and contact. Therefore, constructing a perception system capable of decoding complex deformations is not merely a performance enhancement but the fundamental basis for transforming soft robots from open-loop actuators into intelligent agents with closed-loop cognitive capabilities (*7*–*9*).

Various sensing modalities offer distinct advantages for intelligent systems. Electronic skins based on resistive or piezoelectric arrays offer high-density tactile feedback (*10*, *11*). Liquid metal (*12*) and optical fiber sensors enable flexible strain measurement. Magnetic positioning and inertial measurement units (*13*) excel at capturing rigid-body motion states. Vision-based schemes provide a global field of view for environmental perception. However, transplanting these mature technologies into soft robotics amplifies the conflict between integration and versatility (*14*). This challenge is particularly acute in restricted interventional scenarios where the flexible body requires embedding sensing elements, and space and payload strictly constrain the design. Pursuing sensing versatility and richness often necessitates hardware stacking, while seeking minimalist and easily integrated hardware typically fails to provide sufficient information.

Specifically, on the physical level, discretely distributed sensing units cannot continuously cover the overall structure of a soft robot, resulting in discontinuous spatial sampling (*15*). Complex signal transmission wiring and redundant packaging introduce integration stiffness, which constrains the continuous deformation degrees of freedom inherent to soft structures. High-precision sensing schemes often rely on bulky and complex demodulation equipment, making it difficult to meet the stringent miniaturization requirements of interventional scenarios. External tracking methods also face inevitable line-of-sight occlusion and environmental interference within narrow, curved internal spaces (*16*). Crucially, existing technologies require further advancement at the information level to capture the high-dimensional information density inherent to the infinite degrees of freedom of continuum media (*17*). Therefore, we aim to develop a general perception method suitable for heterogeneous tasks. It must maintain soft body characteristics through minimalist hardware integration while also being capable of encoding and decoding continuous morphological changes during complex interactions.

The optical field is not merely a carrier of energy. Its multiple dimensions including wavelength, phase, intensity, and polarization constitute a vast information phase space (*18*). Optical fiber sensing technology, characterized by micron-level flexible waveguides and inherent electromagnetic immunity, presents a promising solution for complex sensing (*19*, *20*). As mature high-precision optical fiber sensing techniques, fiber Bragg gratings (FBGs) (*21*–*23*) and optical frequency domain reflectometry (OFDR) (*24*, *25*) deliver exceptional measurement accuracy and have been widely deployed in distributed structural health monitoring and industrial sensing. However, these solutions rely on expensive and bulky spectral demodulation equipment and specialty fibers, retrieve deformation via complex demodulation of wavelength shifts, and have highly fixed sensing functions, which greatly restrict their application in low-cost integration scenarios. By contrast, multimode fiber (MMF) serves as a promising alternative. Fundamentally, it is a continuous and complex scattering medium. It supports the transmission of thousands of optical transverse modes (*26*). The intermode interference within it forms a speckle field that is highly sensitive to external physical perturbations (*27*, *28*). This constitutes a high-dimensional information library encoding stress, temperature, and geometric deformation. Therefore, we no longer require complex wavelength demodulation. By simply using a standard image sensor to capture spatial speckle patterns, we can achieve efficient acquisition and processing of high-dimensional modal information. Although deep learning makes decoding these chaotic modes possible (*29*), current MMF sensing solutions still face the dual challenges of hardware limitations and functional limitations. On one hand, most solutions adopt a dual-ended transmission architecture. This forces the fiber to form a loop within spatially restricted interventional tools. This configuration hinders minimally invasive integration. On the other hand, existing research typically treats speckles as static features to train single-task networks. This strong end-to-end constraint leads to poor model generalization capabilities. It also results in excessively high calibration costs. Existing methods still remain at the stage of quantitative sensing of physical quantities such as curvature, displacement, and deflection, thereby failing to satisfy the diverse perception demands of soft robots.

Here, we propose a deep learning–enabled versatile shape perception method for soft robots. In this approach, a minimalist single-ended MMF encodes continuous deformations into high-dimensional speckle fields, which distinct neural decoders subsequently decode into versatile perception outputs tailored to heterogeneous shape tasks. Specifically, on the basis of the shape encoding principle of mode coupling, we simplify the sensor to a standard MMF with a diameter of only ~400 μm. By using a side coupling light injection strategy, we construct a single-ended reflective architecture, effectively eliminating the topological dependence on distal transmission loops, thereby enabling minimally intrusive integration within narrow and tortuous soft structures. Treating the chaotic speckle field as an encodable data stream encoding shape information, we construct reconfigurable neural decoders. Consequently, the system no longer relies on stacked physical sensors but switches perception modes via software, flexibly meeting requirements ranging from discrete state confirmation to continuous shape tracking using a single hardware unit.

Addressing the heterogeneous interaction characteristics of soft robots, we tailored dedicated shape decoding schemes and validated them on three typical platforms. First, we designed a confidence gating network to achieve discrete event confirmation (with >95% confidence) within a ±90° bending range. On a soft gripper, this enabled high-confidence grasp confirmation and curvature determination across 10 classes with >99% accuracy. Second, using a geometric encoding network to learn the smooth dynamic evolution of shape, we realized continuous shape tracking with a ~5-fold enhancement in spatial resolution relative to the sparse sampling, reducing calibration costs. This was demonstrated on a bionic dexterous hand for tracking multijoint complex motions. Third, addressing the safety challenges in visual blind zones, we enabled intuitive three-dimensional (3D) morphological reconstruction [intersection over union (IoU) >0.93] on an interventional surgical robot. By integrating dual-view latent synergy with a retrieval-augmented strategy, we effectively mitigated geometric hallucinations, ensuring physically self-consistent shape perception. This architecture integrates minimalist hardware with versatile decoding capabilities, demonstrating that a single physical carrier, empowered by innovative computational strategies, demonstrates the potential to support the complex perception capabilities required by intelligent robots. This not only provides a versatile perception foundation for next-generation soft robots capable of digital twinning (*30*) but also offers vast potential for functional expansion in the perceptual dimension of future embodied intelligence systems.

## RESULTS

### Minimalist reflective MMF system and versatile neural perception

**Fig. 1.**
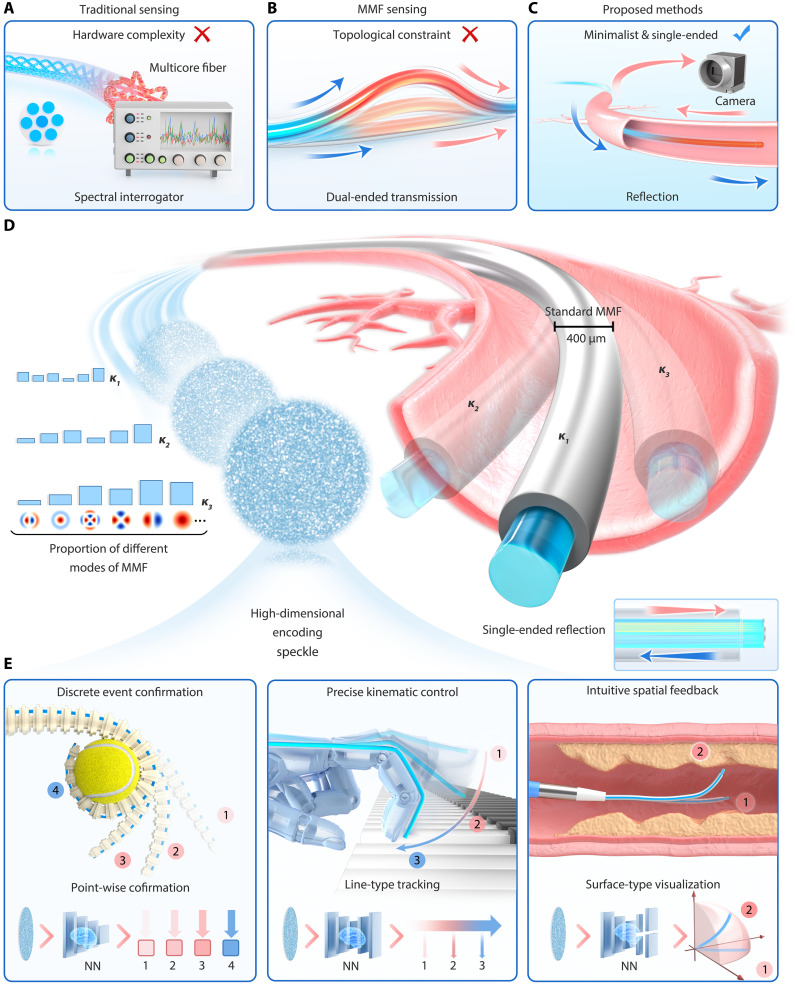
Versatile perception via a minimalist single-ended MMF. (**A**) Hardware complexity in traditional sensing methods. (**B**) Topological constraints of conventional dual-ended MMF sensing, where the transmission loop hinders minimally invasive integration. (**C**) Minimalist architecture of the proposed single-ended integration. The reflective design eliminates distal hardware dependencies, enabling a compact sensing interface. (**D**) Speckle-based shape encoding mechanism within a constrained environment. Deformation modulates internal mode coupling, mapping specific geometric shapes ($K_{1},K_{2},\text{and}\;K_{3}$) to unique high-dimensional speckle fields. (**E**) Versatile neural perception modes decoded from a single interface. The system spans discrete point-wise state confirmation (e.g., stable grasping with soft arms), continuous line-wise shape tracking (e.g., dexterous manipulation), and 3D surface-wise reconstruction (e.g., navigation of vascular interventional robots).

The integration of optical fiber sensing into soft robotics now faces a dilemma between hardware complexity and topological feasibility. As illustrated in Fig. 1A, traditional optical fiber sensing schemes (e.g., FBGs or OFDR) require complex specialty multicore fibers and rely on bulky, expensive interrogators to analyze spectral wavelength shifts, rendering them unsuitable for weight and cost-sensitive embedded applications. However, MMF sensing methods (Fig. 1B), while achieving hardware simplification, adopt a dual-ended transmission structure where the illumination light and shape information light are located on both sides of the sensing segment. This topology physically necessitates the formation of a loop, hindering minimally invasive implantation in interventional scenarios such as vascular branching.

To resolve this conflict, we propose a highly integrated system based on a minimalist single-ended reflective architecture (Fig. 1C). Unlike complex spectral demodulation or dual-ended transmission approaches, we use a side coupling strategy to construct a reflection system based on a single standard MMF. Notably, this efficient coupling strategy stems from our team’s previous exploration and verification in the field of MMF imaging (*31*) and is adapted here to address sensing challenges. This design not only eliminates the need for dedicated interrogators or distal loops but also effectively overcomes the physical limitations of traditional single-ended schemes. Consequently, the system exhibits superior integration and reliability and is better suited for highly integrated applications in extremely space-constrained environments. (The complete optical system setup is described in Materials and Methods and illustrated in fig. S1.)

As shown in Fig. 1D, from a physical perspective, structural deformation of the MMF induces dielectric perturbations, which in turn alter the characteristics of mode coupling and ultimately endow the output speckle field with distinct feature distributions. This process fundamentally conforms to the transmission matrix model of MMFs. Specifically, the input light field can be decomposed into a linear superposition of the *N* guided modes supported by the fiber. As these modes propagate, they undergo continuous mutual coupling and weight reorganization, and their complex amplitudes evolve progressively along the fiber axis, thus forming a final output speckle field expressed as

$$I\left(r,\varphi \right)={|E_{\text{out}}\left(r,\varphi \right)|}^{2}={\left|\sum_{\mu =1}^{N}c_{\mu }\left(L\right)E_{\mu }^{\left(t\right)}\left(r,\varphi \right)\right|}^{2}$$ (1)

Here, $E_{\mu }^{\left(t\right)}\left(r,\varphi \right)$ denotes the transverse electric field distribution of the μth mode, and *c*_μ_ represents the coupling coefficient of this mode. Furthermore, when the fiber undergoes arbitrary spatial deformations in 3D space, distributed tensile and compressive stresses are generated between its inner and outer layers. Through the photoelastic effect, such stresses are converted into asymmetric perturbations of the dielectric tensor **ε**, thereby effectively encoding the 3D morphological information of the fiber into the mode evolution process. Considering the influence of global morphological perturbations, the mode propagation equation simplifies to

$$\frac{dc\left(z\right)}{dz}=-j\left(\beta +K_{\text{deformation}}\left(\varepsilon \right)\right)c\left(z\right)$$ (2)

where β *=* diag(β_1_, β_2_, …, β*_N_*) is the diagonal matrix of ideal propagation constants, and the *N × N* matrix ***K***_deformation_ represents the deformation-induced mode coupling matrix. The evolved mode amplitude vector ***c***(*z*) accumulates various perturbation information along the fiber path, and each specific shape of the MMF (i.e., the shape labeled as ***K***_1_, ***K***_2_, and ***K***_3_ in the figure) corresponds to a unique set of fiber mode weights, thus generating a distinct speckle intensity distribution. (A detailed derivation of the physical link between geometric deformation, dielectric perturbation, and mode coupling is provided in section S1.) In contrast to conventional algorithms relying on discrete point interpolation, this global optical response enables neural networks to perceive the continuous deformation of the fiber with high fidelity. Crucially, this chaotic speckle field acts as a high-dimensional information carrier, which contains far richer structural encoding information than simple spectral or intensity metrics alone.

Building on this minimalist reflective system, we propose a versatile perception method that decouples sensing capabilities from hardware complexity. Crucially, we use the single-ended MMF as a unified physical interface that encodes continuous deformations into high-dimensional speckles. Unlike traditional singular-output sensors, we use distinct neural decoders to decode these chaotic signals from the same hardware into versatile perception modes tailored to heterogeneous tasks, spanning discrete point-wise, continuous line-wise, and 3D surface-wise perception capabilities (Fig. 1E).

First, for scenarios requiring reliable discrete decision-making, we use a point-wise confirmation strategy. As demonstrated by the stable grasping of a tennis ball, this mode prioritizes decision certainty, allowing the robot to confirm specific contact events or critical operational nodes while effectively filtering out transitional noise. Second, for tasks demanding high-precision kinematic control, we introduce a line-type tracking approach. This capability is essential for applications like the dexterous manipulation of bionic fingers, such as playing the piano, where the system must continuously resolve smooth, microscale angular changes to ensure fluid and coordinated motion. Last, for medical navigation requiring intuitive spatial feedback, we realize surface-type reconstruction. By reconstructing the 3D morphology of the instrument within the blind zones of surgical environments, this mode provides the in situ digital twin awareness necessary for safe operation in confined anatomical spaces. Those versatile perception modes enable a single fiber to flexibly adapt to diverse perception requirements, providing a versatile solution for intelligent soft robots by fully exploiting the rich, multidimensional physical information embedded within the speckle field to meet the dynamic needs of unstructured environments. (Evaluation metrics and task-specific network architectures are defined in Supplementary Text, with structural details provided in fig. S2.)

### Discrete state confirmation via confidence-gated neural networks

To ensure maximum decision reliability, we first deploy a classification-based decoder to address discrete event confirmation. Reliable state confirmation serves as the cornerstone for precise sequential decision-making in intelligent soft robots. Critical tasks, including grasping verification or assembly alignment, demand absolute certainty to authorize subsequent actions (*32*–*34*). To achieve this, we developed a discrete confirmation strategy that prioritizes decision reliability via a sparse calibration approach.

**Fig. 2.**
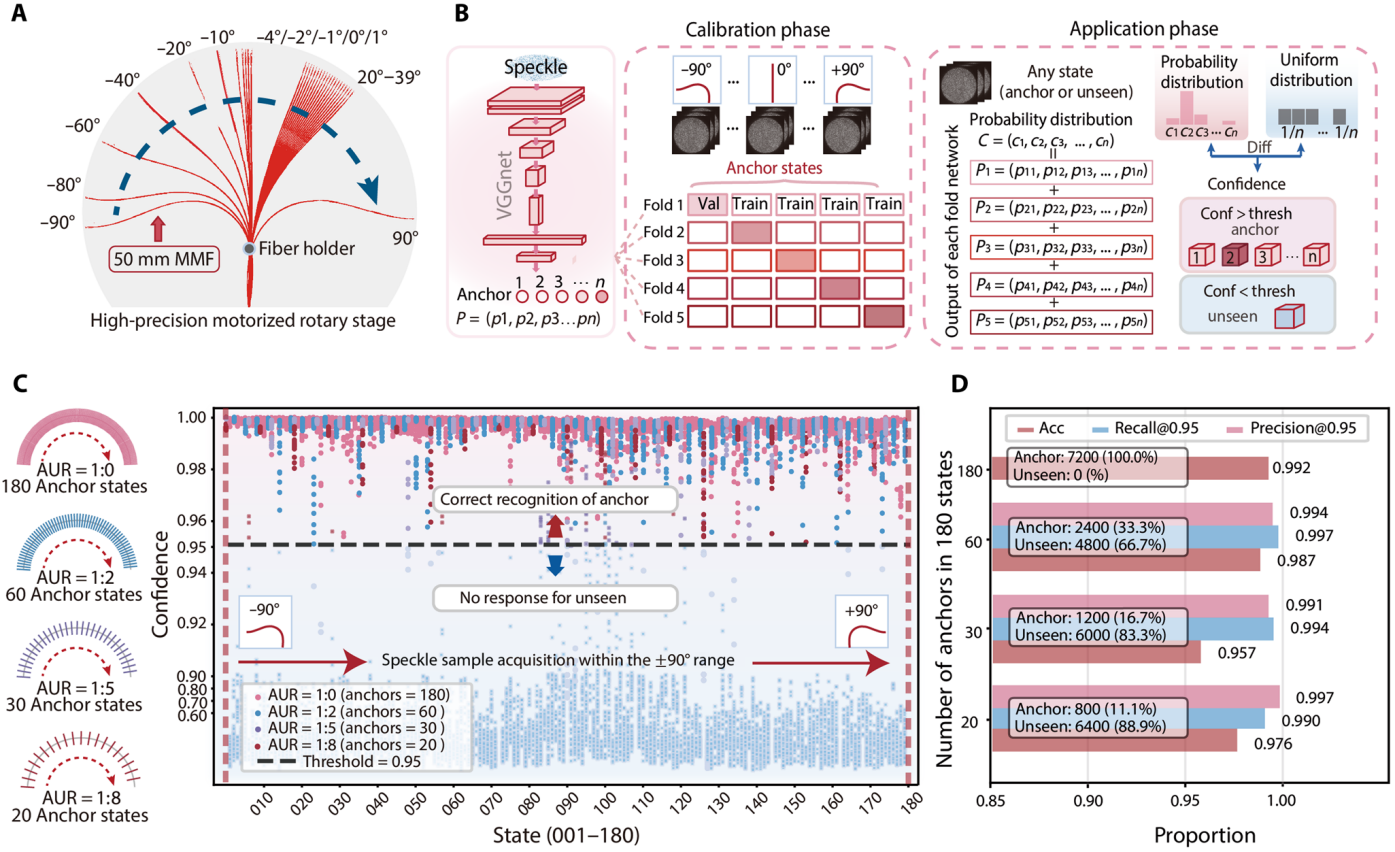
Precise discrimination of discrete anchor states and performance analysis. (**A**) Data acquisition system for MMF distal shape, controlled by a high-precision rotation stage. (**B**) Fivefold cross-validation architecture based on a VGG network and the confidence-driven soft voting strategy. The calibration phase uses discrete anchor data for neural network training, while the application phase tests with all collected data containing both anchor and unseen points. States are correctly classified into an anchor category when confidence exceeds the 0.95 threshold; the system withholds response for low-confidence inputs. (**C**) Scatterplot distribution of confidence scores for all samples under four different AUR settings, with data collected continuously across the ±90° range. Colors indicate AUR = 1:0, 1:2, 1:5, and 1:8, respectively. (**D**) Comprehensive system performance evaluation: including anchor classification accuracy, recall, and precision for unseen states.

We fixed a 50-mm sensing segment to a high-precision rotation stage to simulate continuous morphological changes. The shape of MMF was quantified via the distal tip angle (Fig. 2A). (Data acquisition protocols and preprocessing details are provided in Supplementary Text, fig. S3, and movie S1.) As visualized by the superimposed red curves, the fiber exhibits distinct geometries across a wide ±90° range. From this angular range, specific discrete angles were calibrated as anchor states for training, while the vast continuous intervals between them were designated as unseen states that must be effectively filtered.

To process the high-dimensional speckle information, we used a visual geometry group (VGG)-based network architecture as shown in Fig. 2B. It effectively compresses the global light field distribution and its spatial correlations into a compact feature vector. Simultaneously, this process isolates deformation-sensitive features from background noise. To ensure robust generalization and prevent overfitting, we implemented a fivefold cross-validation strategy, where the system aggregates probability distributions *P_n_* from five independent subnetworks via a soft voting mechanism. Crucially, we constructed a confidence gating mechanism by quantifying the certainty of prediction. We define the confidence metric *C*(*c*_1_, *c*_2_, *c*_3_, …, *c_n_*) using the Kullback-Leibler (KL) divergence between the predicted probability distribution and a uniform distribution. The system outputs a specific anchor state only if this confidence score exceeds a stringent threshold of 95%. Otherwise, the input is classified as an uncalibrated state. This ensures that the system operates with near-zero false positives during uncertain transitions. Discrimination relies on the actual learned distribution of each anchor state, rather than a single ideal mathematical point. Consequently, the system can robustly adapt to inherent state fluctuations and jitter in practical applications.

We rigorously evaluated the system’s selectivity by challenging it with varying densities of valid states, quantified by the anchor-to-unseen ratio (AUR). A lower AUR implies that the system must identify a few specific functional states amidst extensive continuous perturbations. The scatterplot in Fig. 2C visually confirms the robustness of this mechanism: Regardless of the AUR configuration, the confidence scores exhibit a distinct stratification. Valid anchor samples (top cluster) consistently concentrate near the confidence ceiling (1.0), while the vast majority of unseen samples (bottom cluster) are effectively suppressed below the 0.95 threshold. Quantitative results (Fig. 2D) demonstrate that even at an extreme ratio of AUR = 1:8, the system maintains high stability. The classification accuracy for anchor states reached 98.7%, while both the recall and precision for rejecting unseen states exceeded 99%. This proves that our sparse calibration strategy does not compromise performance; rather, it demonstrates the system’s capacity to selectively recognize functional states amidst extensive continuous interference.

**Fig. 3.**
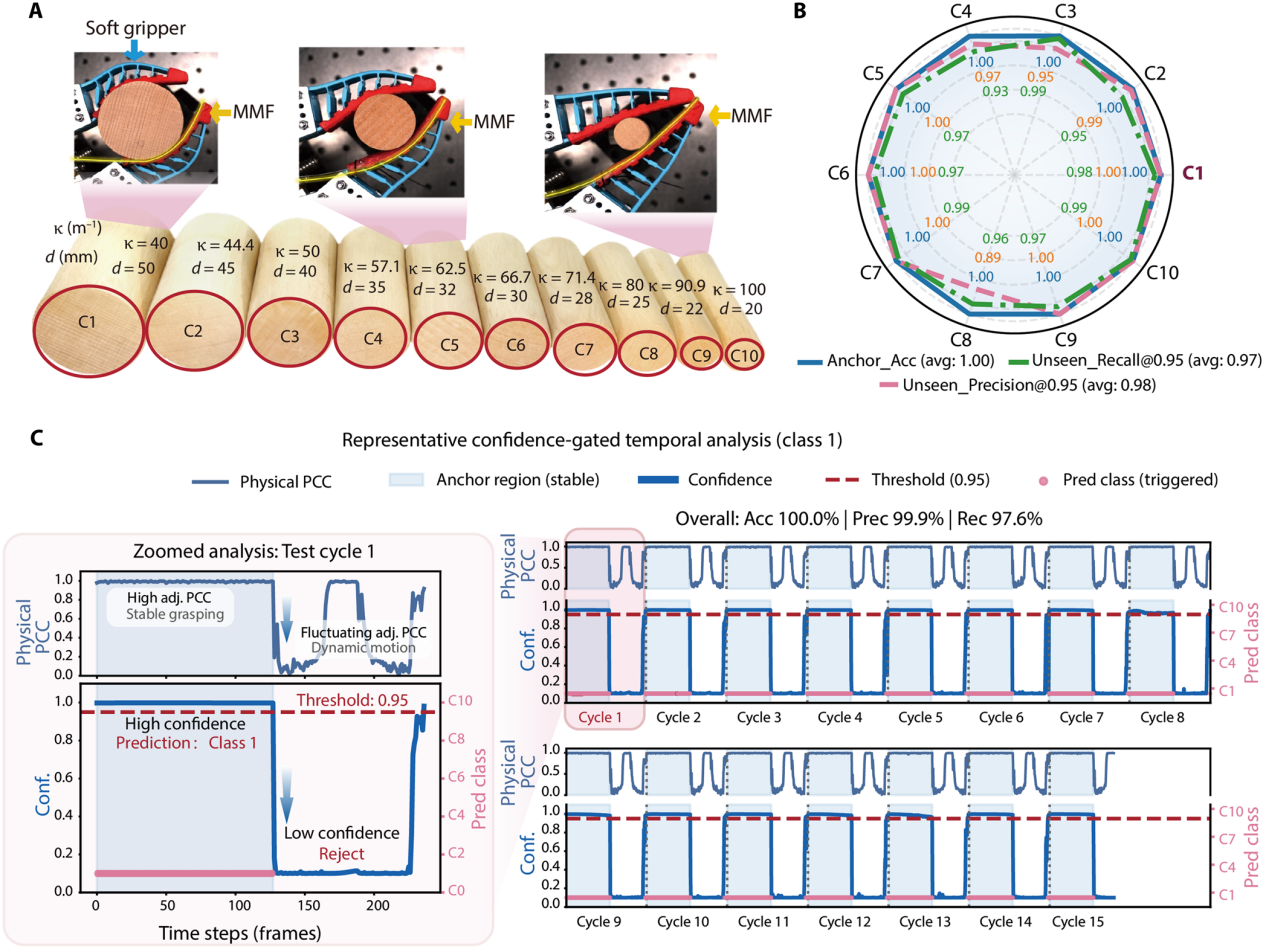
Dynamic discrete state confirmation applied to a soft gripper. (**A**) Soft gripper experimental setup. The fiber-integrated robot interacts with 10 cylindrical rods, using deformation-induced speckle changes for state encoding. (**B**) Holistic performance evaluation. The radar chart confirms that the perception system adapts well to the soft gripper’s diverse grasping topologies, achieving high accuracy, precision, and recall across all curvature classes. (**C**) Representative confidence-gated temporal analysis (class 1). The left (cycle 1) illustrates that the neural network becomes cognitively confident and triggers classification only when the soft gripper is in a physical steady state (high PCC) while suppressing output during dynamic motion (low PCC). The right (15-cycle sequence) validates the long-term reliability of this gating network, showing that trigger signals remain highly consistent without drift under repetitive operation.

To validate the proposed strategy in a realistic robotic scenario, we integrated the MMF into a soft gripper and conducted a sequential grasping experiment involving 10 cylindrical rods with distinct curvatures (*κ* = 40 to 100 m^−1^, labeled C1 to C10, as shown in Fig. 3A). Unlike the previous setup where the ground truth was rigidly defined by the absolute coordinates of a motorized stage, the grasping process of soft robots involves continuous flexible deformation that cannot be absolutely quantified, resulting in a lack of the ground truth reference in absolute world coordinates. To address this, we used a data-driven labeling strategy where the stability of the speckle field, quantified by the adjacent frame Pearson correlation coefficient (PCC), serves as the criterion for defining grasping events. Consequently, only intervals exhibiting high optical stability were labeled as valid anchor states, while dynamic transition phases were designated as unseen states.

In Fig. 3B, we evaluated the holistic performance of this strategy using a radar chart. The results demonstrate that the system maintains exceptional consistency across all 10 curvature classes. The accuracy (correct identification of the specific rod), precision (reliability in avoiding false triggers during motion), and recall (sensitivity in detecting every valid stable grasp) for every class are tightly clustered in the high-performance region (>0.95). This uniform performance envelope confirms that the system is robust against topological variations and ensures reliable operation across the full curvature range of 40 to 100 m^−1^. Figure 3C presents a detailed temporal analysis of the first class. As shown in the single-cycle analysis (cycle 1) on the left, the system exhibits substantial synchronization between the physical grasping state and prediction confidence. Specifically, when the soft gripper reaches a steady state (PCC approaches 1.0), the neural network’s output confidence simultaneously climbs to 1.0, thereby activating the classification output. Conversely, during dynamic motion phases, rapid fiber deformation causes severe PCC fluctuations; in response, the model actively suppresses output by maximizing uncertainty, causing the confidence score to drop sharply and thus preventing spurious triggers. The 15 continuous test cycles shown on the right further validate the temporal robustness of this mechanism. Under prolonged repetitive mechanical stress, the system consistently maintains this responsive pattern without signal drift or degradation. (See fig. S4 for temporal analyses of other classes.)

### Continuous super-resolution tracking via geometric priors

While discrete state confirmation provides reliable determination for specific operational nodes, sophisticated tasks such as dexterous manipulation of bionic fingers require continuous high-resolution tracking of deformations (*35*). These scenarios demand a seamless stream of geometric data rather than binary indicators. However, achieving this faces a fundamental physical resolution limit.

**Fig. 4.**
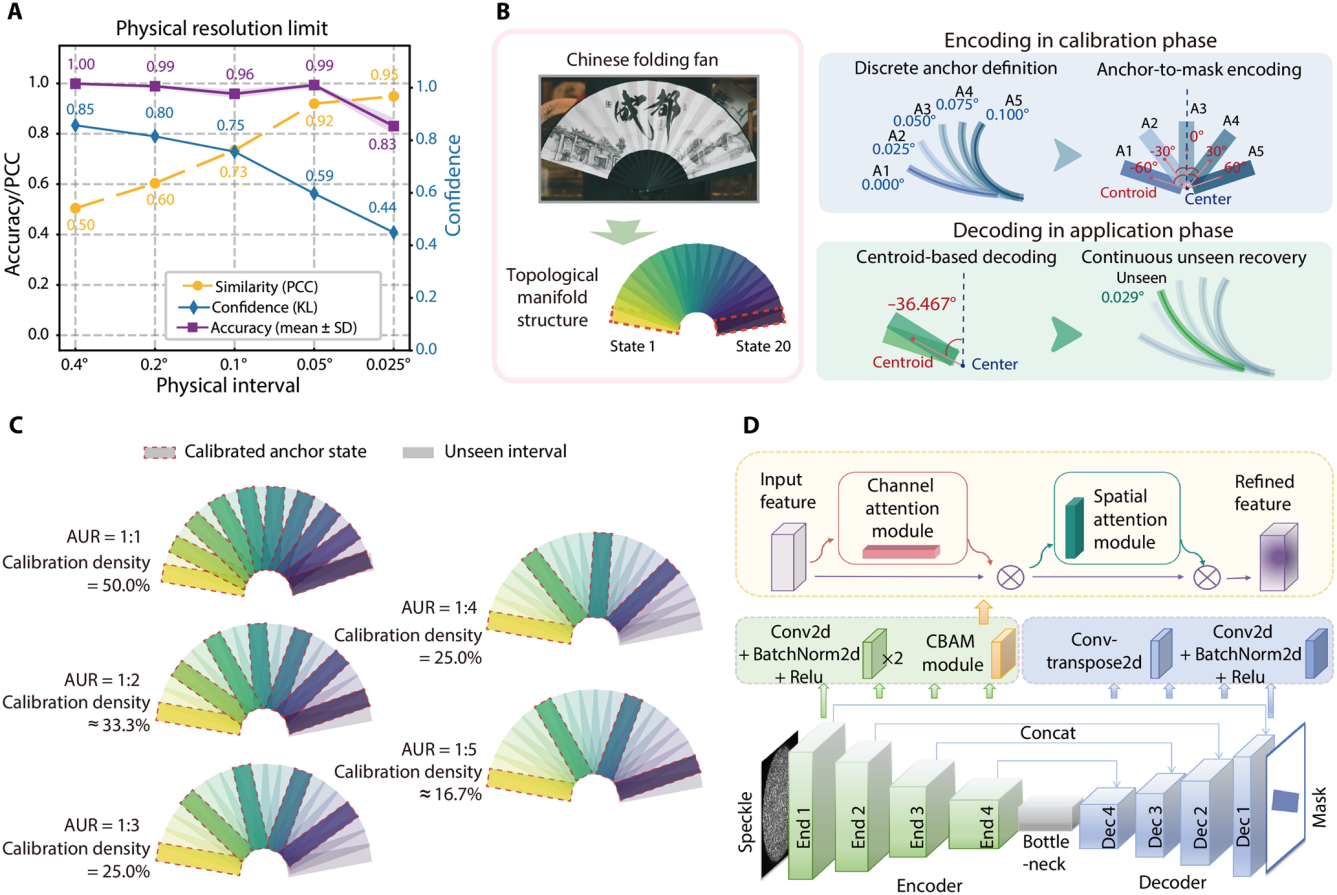
Overcoming physical resolution limits via structured geometric encoding. (**A**) Multidimensional analysis of the resolution limit. The crossover between the classification accuracy (purple line) and the rising interclass PCC (yellow line) highlights the blind zone of hard classification at micro-intervals. The diminishing KL divergence (blue line) visualizes the decay of network confidence. (**B**) The structured geometric encoding strategy inspired by a folding fan topology (photo of the folding fan from Unsplash by T. Su, Unsplash license: https://unsplash.com/photos/C8GL82YeSmU). Physical angles of MMF are mapped to a 2D sector mask, where “fan ribs” represent discrete anchor states and the fan surface represents continuous unseen intervals. This converts single-value prediction into a collective spatial estimation task, enabling the network to learn the continuous deformation manifold. (**C**) Visualization of the sparse calibration protocols with varying AURs from 1:1 to 1:5. (**D**) Architecture of the CBAM-integrated UNet used for feature refinement and mask regression.

To systematically quantify this limitation, we conducted a multidimensional analysis correlating physical similarity with network uncertainty across decreasing deformation intervals (from 0.4° to 0.025°). As illustrated in Fig. 4A, a critical crossover phenomenon emerges: As the interval narrows, the interclass PCC (yellow line) rises sharply toward 1.0, indicating that signal variations are increasingly submerged in system noise. Simultaneously, the classification accuracy (green line) declines sharply, distinctly intersecting with the PCC curve. Furthermore, the network’s internal uncertainty (quantified by the KL divergence, blue line) exhibits a rapid decay, diminishing as the interval shrinks. This convergence of high physical similarity and low prediction confidence demonstrates that forcing a discrete categorical decision onto continuous microdeformations is mathematically ill-posed under low signal-to-noise ratio conditions.

To bypass this bottleneck without hardware complexity, we shifted the paradigm from discrete classification to continuous regression by proposing a structured geometric encoding strategy (Fig. 4B). Instead of predicting discrete labels, we map the continuous physical angle to a topologically continuous 2D sector mask. Unlike the discrete strategy where unseen states imply rejection, here, we redefine unseen as interpolation intervals on a continuous manifold. As visualized, the “fan rib” angular positions correspond to calibrated anchor states, while the expanding “fan surface” represents the continuous deformation space. During training, the network explicitly learns the nonlinear mapping between speckles and anchor masks. During inference, the continuous angle is regressed via centroid decoding of the predicted mask. This introduces a powerful geometric prior: The topological continuity of the fan’s expansion forces the neural network to learn the smooth evolution of the deformation manifold rather than memorizing discrete textures. Moreover, centroid decoding expands the solitary algebraic constraint of an angle value into a collective spatial constraint across thousands of pixels, using statistical correlations to effectively filter out local prediction noise.

We defined the experimental validation under varying calibration densities, as outlined in the AUR configuration schematic (Fig. 4C). To implement the speckle-to-mask mapping, we used a UNet architecture integrated with convolutional block attention modules (CBAM) (Fig. 4D). Unlike standard convolutions, the CBAM blocks adaptively refine features via a dual-attention mechanism: Channel attention selectively weights effective optical mode channels (focusing on what features are relevant), while spatial attention suppresses background noise to focus on the deformation-sensitive boundaries of the fan ribs (focusing on where the features are located).

**Fig. 5.**
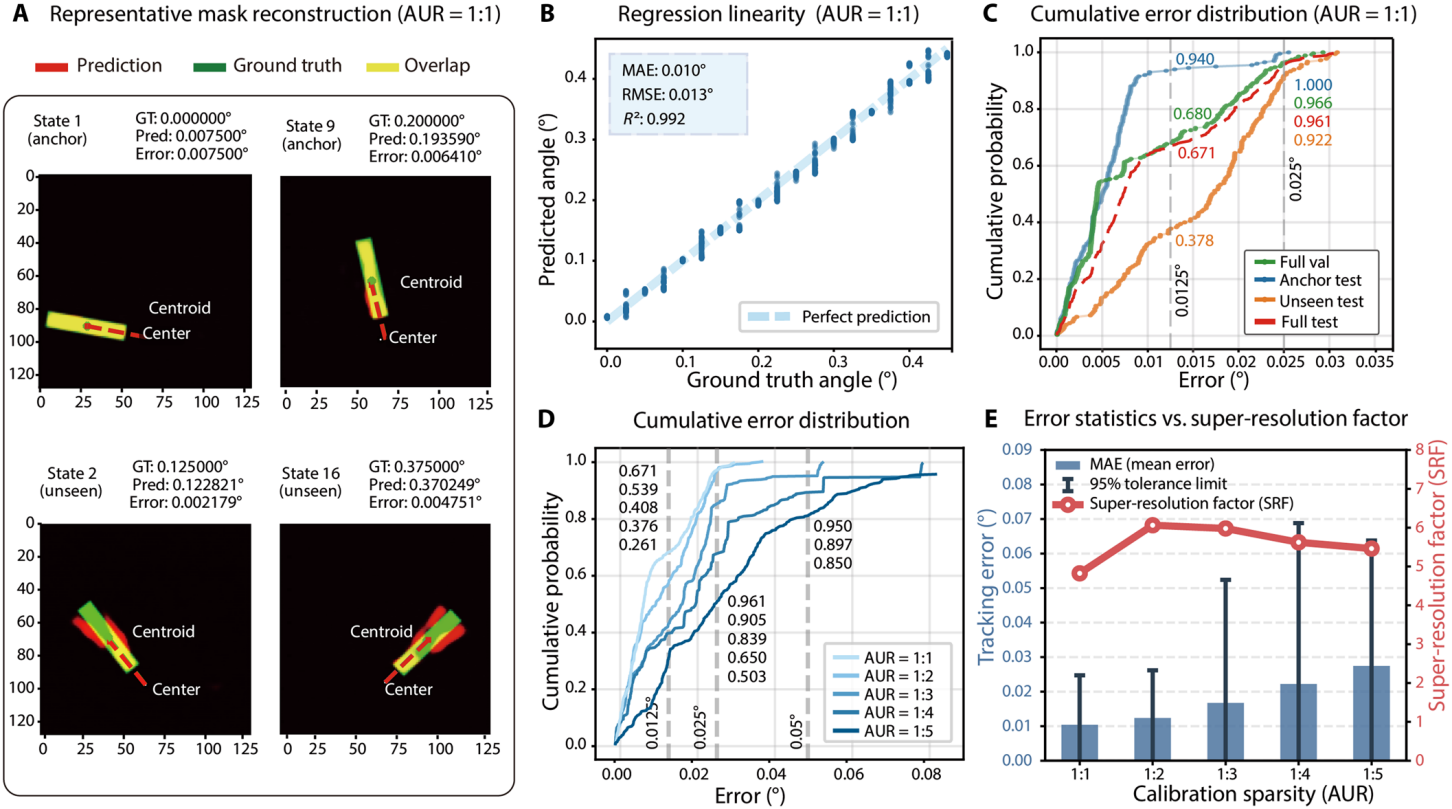
Performance evaluation of super-resolution tracking under sparse calibration. (**A**) Representative mask reconstruction results. The overlay of prediction (red) and ground truth (GT, green) demonstrates robust centroid alignment even when morphological reconstruction is slightly imperfect. (**B**) Linearity analysis showing the system achieves a mean absolute error (MAE) of 0.010°, effectively bridging the 0.05° training interval (*R*^2^ > 0.99). (**C**) Cumulative error distribution (CDF) confirming 96.1% accuracy within the 0.025° tolerance window. (**D**) Comparative CDFs across varying densities (AUR 1:1 to 1:5) illustrate interpolation robustness, with minimal performance degradation even as data is reduced by 80%. (**E**) Statistical summary of perception precision. The bar chart (left axis) displays the MAE and 95% tolerance error limits, showing high stability. The red curve (right axis) quantifies the super-resolution factor (SRF), defined as the ratio of the calibration interval to the MAE. The rising SRF curve demonstrates that the geometric priors effectively compensate for data sparsity.

The reconstruction is visualized in Fig. 5A, where red regions represent the predicted mask, green indicates the ground truth, and yellow denotes the overlap. Examining the reconstruction of unseen state reveals that while minor morphological mismatches exist, the centroid of the predicted area remains robustly aligned with the true angle. (For more detailed reconstruction examples, please refer to figs. S5 and S6 and movie S2.) This indicates that the centroid decoding method effectively filters pixel-level high-frequency noise, ensuring high angular precision through collective pixel statistics.

We evaluated this strategy under a sparse calibration setup (AUR = 1:1, training interval of 0.05°, and testing interval of 0.025°). The linearity results in Fig. 5B demonstrate a subgrid resolution capability. Although the system was not trained on the intermediate 0.02° states, the predicted angles follow the diagonal, achieving a mean absolute error (MAE) of 0.01°. This precision, surpassing the intrinsic 0.05° resolution of the training grid, confirms that the model successfully learns the continuous geometric manifold rather than merely memorizing discrete states. The cumulative error distribution (Fig. 5C) further quantifies this performance. Within the 0.025° tolerance window, the system achieves a 96.1% average accuracy (100% for anchors and 92.2% for unseen states). Notably, this regression-based strategy yields higher reliability (96.1%) using only half the calibration data compared to the dense calibration classification model (83%).

To evaluate the limits of mask interpolation, we relaxed the calibration density from an AUR of 1:1 to 1:5. Figure 5D illustrates the accuracy decay as sparsity increases. The system avoids substantial performance degradation, maintaining a coherent response curve. As shown in Fig. 5E, the blue bars indicate that the MAE increases from 0.010° to 0.027° as data become sparser. However, the super-resolution factor (SRF), defined as the ratio of the training interval to the achieved resolution (MAE), remains consistently around 5. This implies that despite the increase in absolute error with wider training intervals, the model consistently resolves angular details five times finer than the provided calibration grid. This confirms the robustness of the learned mask structure, allowing for a substantial reduction in data acquisition volume while maintaining a relative precision advantage.

**Fig. 6.**
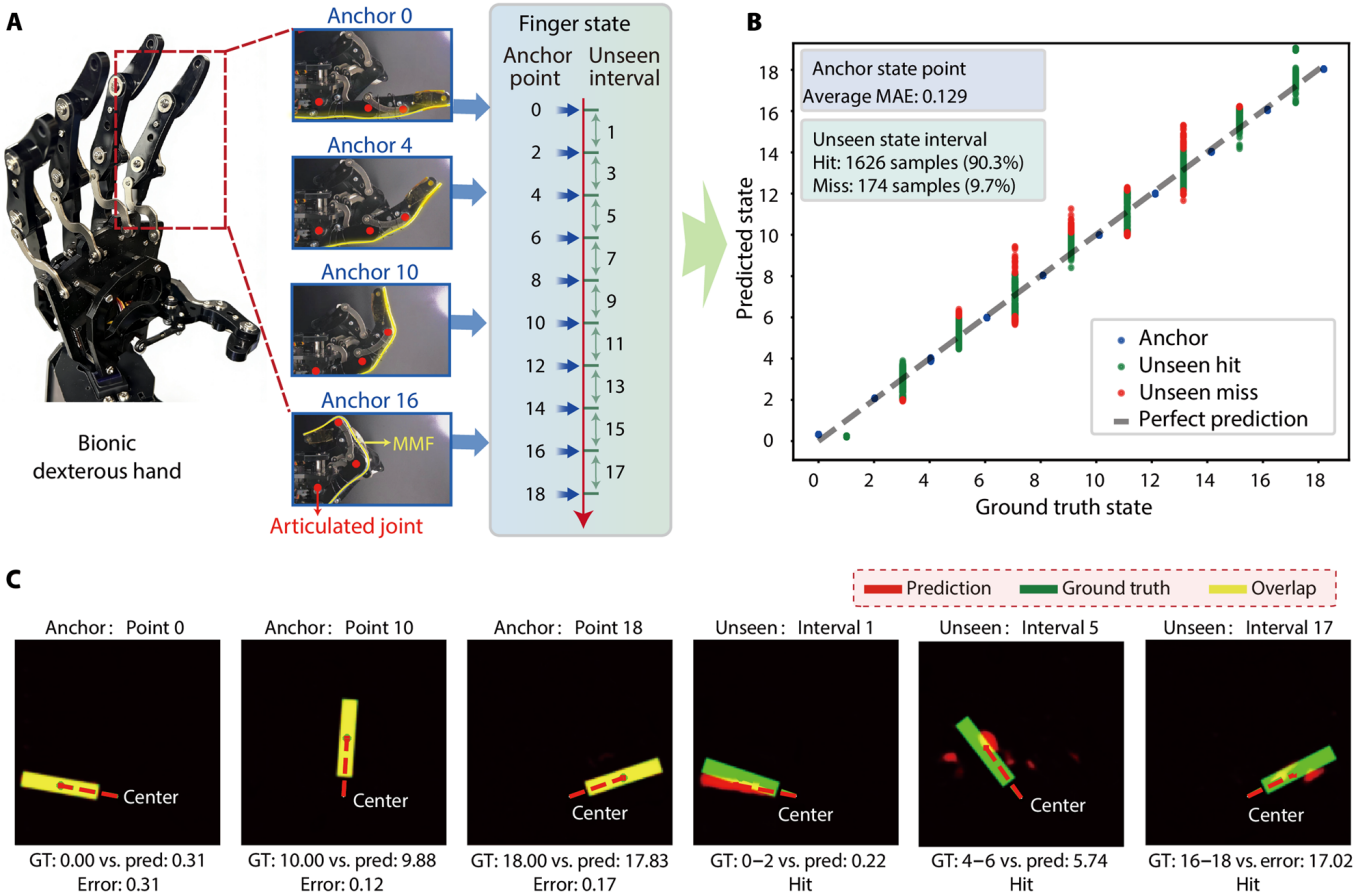
Continuous pose perception performance on a bionic dexterous hand. (**A**) Definition of the test space: 10 discrete anchor points along the index finger’s trajectory and the nine unseen intervals between them. (**B**) Dual-metric quantitative analysis. The system achieves a low MAE of 0.129° on anchors (limited primarily by mechanical jitter) and a 90.3% interval hit rate on unseen regions, confirming robust generalization. (**C**) Visualized decoding results.

In the final validation phase, we examined the adaptability of the proposed encoding strategy on a bionic dexterous hand. This device features a complex multijoint structure characterized by nonlinear deformation patterns and strong stress gradients. We selected 10 discrete states along the index finger’s motion trajectory as anchor points (Fig. 6A), covering the full range from complete extension to maximum flexion. To rigorously evaluate system performance across the entire motion space, we designated the continuous deformation zones between adjacent anchors as unseen intervals.

Given the challenges in acquiring absolute ground truth during continuous dynamic transit, we adopted a dual-metric evaluation strategy. For the calibrated anchor points, we assessed absolute precision using a hold-out test set; for the uncalibrated unseen intervals, we evaluated the “interval hit rate”—a metric determining whether the predicted angle logically falls within the bounds of the corresponding adjacent anchors. Experimental results (Fig. 6B) reveal that the MAE for anchor states is 0.129. Notably, this residual error largely reflects the intrinsic mechanical jitter of the robotic system rather than algorithmic inaccuracy, suggesting that the perception precision approaches the mechanical repeatability limit. Meanwhile, the unseen states achieved a 90.3% interval hit rate, indicating that most of the continuous predictions are correctly mapped to their true deformation zones without appreciable deviation. To provide an intuitive verification of this perception stability, Fig. 6C presents the visualized decoding results for representative states. These results confirm the generalization capability of our encoding method even under the complex mechanical coupling conditions of multijoint mechanisms, providing a robust pose feedback foundation for the high-precision closed-loop control of bionic dexterous hands.

### Intuitive 3D morphological reconstruction via retrieval-augmented generation

Building on the discrete state confirmation and continuous kinematic tracking that effectively support closed-loop control in soft robotics, clinical scenarios involving high-risk medical interventions demand a higher dimension of “safety awareness” through visualization (*36*). Complex endovascular procedures, such as pulmonary endarterectomy, require instruments to navigate deep into pulmonary artery branches with average diameters of only 2.0 to 4.0 mm. In these scenarios, beyond precise motion control, surgeons require intuitive holistic morphological awareness within the visual blind zone to prevent the flexible instrument from undergoing unexpected large-scale deformations that could damage the vessel walls. To bridge this gap from kinematic parameter monitoring to 3D spatial morphological perception, we propose a shape perception strategy based on retrieval-augmented generation (RAG). This strategy aims to reconstruct a physically self-consistent 3D instrument skeleton by leveraging the rich structural information encoded within 2D fiber speckles, thereby enabling transparent, radiation-free navigation in deep lesion areas.

**Fig. 7.**
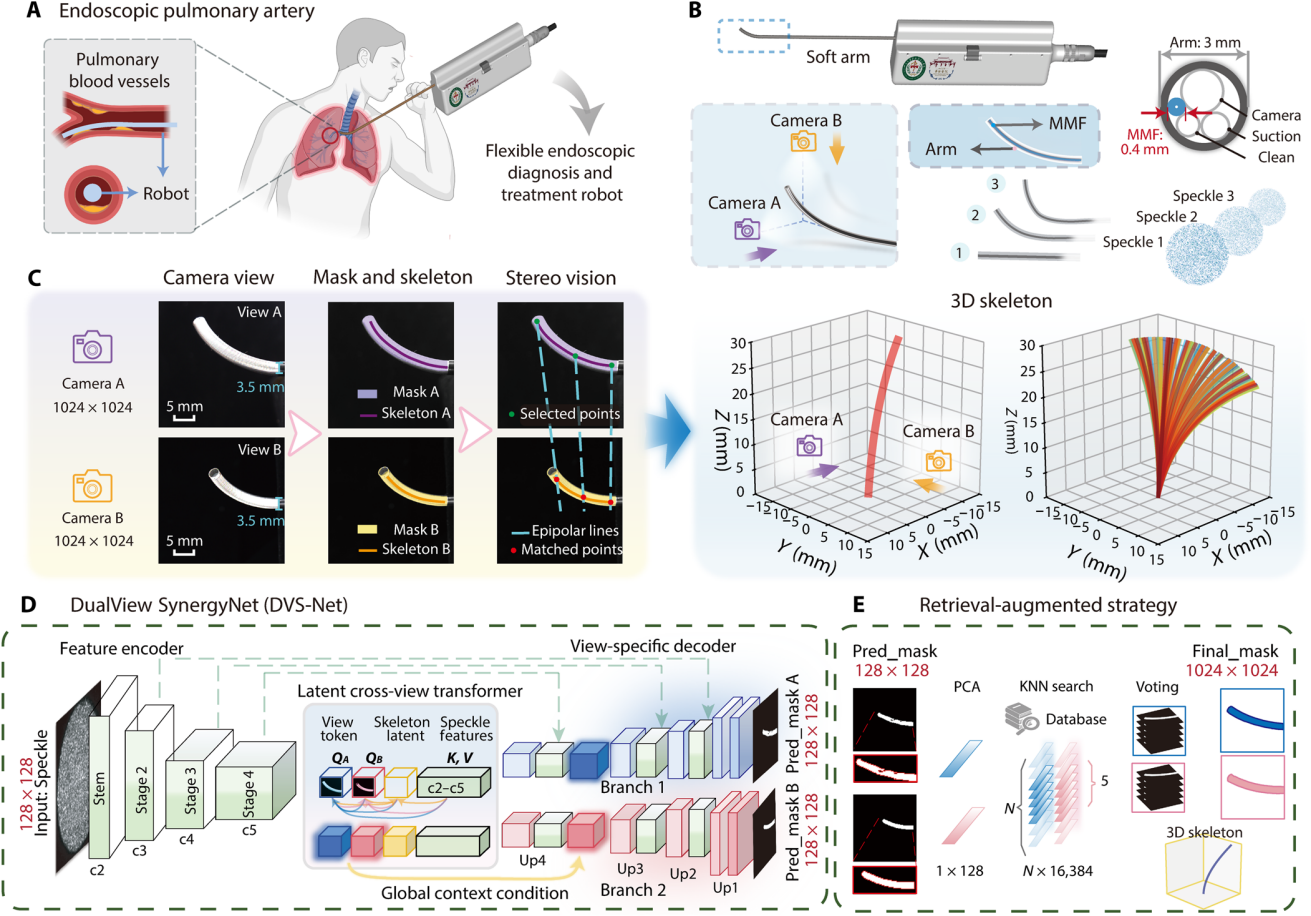
Intuitive 3D shape perception via retrieval-augmented dual-view synergy. (**A**) Clinical application scenario [Created in BioRender. He, Z. (2026) https://BioRender.com/c9ek28y; created in BioRender. He, Z. (2026) https://BioRender.com/baa9f1o]. Schematic of flexible endoscopic robot in a pulmonary thromboendarterectomy scenario. (**B**) Soft robotic arm integrated with single-ended MMF and dual-view visual calibration system, using two orthogonal cameras to calibrate every morphological state of the flexible arm via speckle fields. (**C**) 3D skeleton generation. Masking and skeleton extraction are performed on 1024 × 1024 dual-view calibration images, using epipolar geometry methods to extract high-precision 3D skeleton information from orthogonal views. (**D**) DualView SynergyNet (DVS-Net) architecture, comprising feature extractors, latent space cross-view transformer, and view-specific feature decoders. (**E**) RAG workflow. Coarse masks (Pred_mask A and B) predicted by the DVS-Net undergo principal components analysis (PCA) feature extraction followed by a KNN search (*K* = 5) in the database. The final 3D skeleton reconstruction is based on refined masks (Final_mask A and B) generated via voting from the retrieved top-K samples, ensuring physical fidelity.

We integrated this system into a robotized flexible dissector prototype (Fig. 7A), which has been validated through ex vivo porcine lung experiments and holds potential for pulmonary thromboendarterectomy applications. To provide a reliable physical reference for the fiber optic sensing model, we constructed a visual calibration system based on dual orthogonal views (Fig. 7B). By applying epipolar geometry principles to perform stereo matching on the captured high-resolution orthogonal video streams (Fig. 7C), we demonstrated that precise 3D skeletal information can be recovered solely from orthogonal 2D binary (1-bit) images. On the basis of this principle, we constructed a prior database of high-resolution masks containing physically valid morphologies. It is important to emphasize that external cameras are used strictly for data acquisition and database construction during the offline training phase; intraoperative nonvisual perception relies exclusively on optical fiber signals.

Algorithmically, to map 2D speckles to 3D spatial morphologies, we designed the DualView SynergyNet (DVS-Net) (Fig. 7D). Although the input from a single fiber consists of 2D speckle features, morphological reconstruction requires generating geometric information for two orthogonal perspectives. To address this, we introduced a latent cross-view transformer module (*37*) to achieve feature synergy between the dual views. Specifically, we defined learnable view tokens (*Q_A_* and *Q_B_*) as query vectors to retrieve geometry-related information specific to each perspective from the 2D speckle features (*K* and *V*) extracted by the encoder. More critically, to constrain geometric consistency between the two views, we embedded shared skeleton latent tokens within the network. These latent tokens force the network via attention mechanisms to learn the intrinsic structure of the object in 3D space, rather than merely fitting two independent planar projections, thereby generating preliminary coarse masks.

However, constrained by the computational bottleneck of end-to-end networks, the resolution of directly generated masks is typically low (128 × 128), which risks losing high-frequency details or producing geometric hallucinations that violate physical constraints when processing minute deformations. To mitigate this risk and break through resolution limits, we introduced an RAG inference workflow (Fig. 7E). In this process, the low-resolution coarse mask generated by DVS-Net serves not as the final output but as a retrieval index. The system extracts the principal components analysis (PCA) feature vector (1 × 128) of this mask and performs a K-nearest neighbor (KNN) search within the prebuilt morphological prior database. The system then retrieves the top-K most similar high-resolution real physical samples (1024 × 1024) and generates the final refined mask (Final_mask) through a weighted voting mechanism, subsequently reconstructing a high-precision 3D skeleton. This strategy fundamentally transforms an unconstrained generation task into a sample retrieval task strictly bound by physical laws. This not only resolves the blurring issue associated with low-resolution generation but also ensures that the reconstructed 3D morphology remains physically plausible and geometrically self-consistent, even when the instrument undergoes microjitters or extreme deformations.

**Fig. 8.**
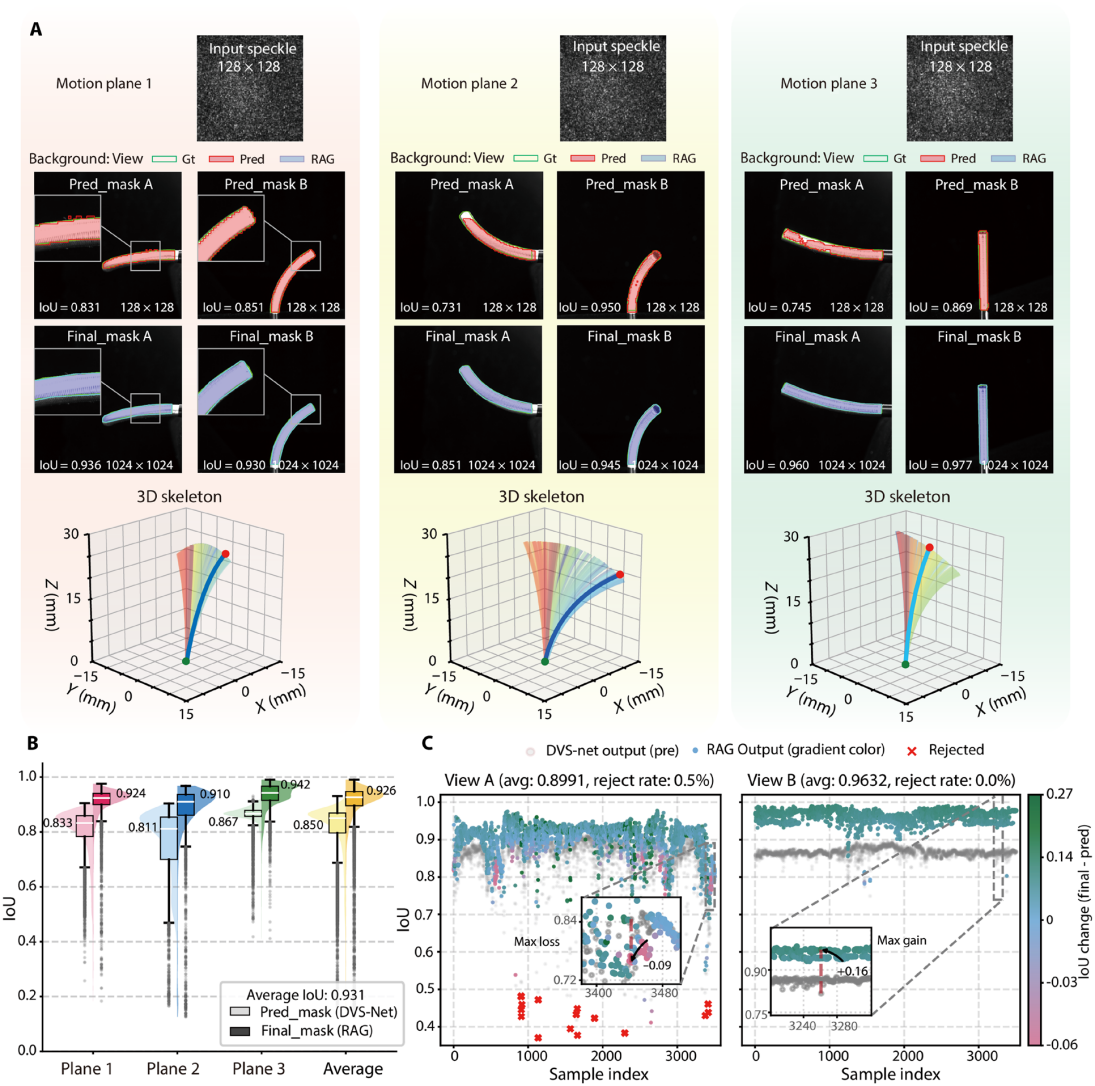
Super-resolution reconstruction and outlier rejection via RAG. (**A**) Qualitative comparison of reconstruction fidelity across three motion planes, which represent three typical axial torsion states of the MMF. The top row displays the raw input speckle fields. The middle row shows the coarse masks predicted by DVS-Net (red outlines), exhibiting aliasing artifacts due to resolution limits. The bottom row presents the refined masks after RAG (blue fills), demonstrating smooth boundaries and high alignment with the ground truth (green outlines). The corresponding 3D skeletons (bottom) visualize the recovered spatial trajectories. (**B**) Quantitative box plots comparing IoU distributions before (light boxes) and after (dark boxes) RAG enhancement. The strategy consistently shifts the performance distribution upward, achieving a final average IoU of 0.931 across all planes. (**C**) Scatterplot analysis of the outlier rejection mechanism and IoU evolution. Gray dots represent the initial DVS-Net output, while colored dots represent the RAG output, with the color gradient indicating the magnitude of IoU change (gain/loss). Red crosses mark samples rejected due to low confidence (<50% overlap). Insets highlight specific examples of maximum IoU gain (+0.16) and minor loss (−0.09), verifying the overall stability of the retrieval strategy.

To validate the efficacy of this strategy, we conducted comprehensive reconstruction tests using a flexible arm with a deformable length of 30 mm and a diameter of 3 mm across three trajectory planes with distinct motion characteristics. Notably, these three planes represent three typical axial torsion states of the MMF. By successfully reconstructing the morphology across these distinct planes, we can demonstrate that the network has an effective morphological representation capability for various shape signals, including bending and torsion. Here, the IoU is used as the core metric to quantify the pixel-level overlap consistency between the predicted mask and the true physical morphology. As shown in the reconstruction results in Fig. 8A, although DVS-Net can recover the general morphology of the instrument, its direct output predicted mask inevitably suffers from pixel-level discontinuities and aliasing artifacts due to the aggressive upsampling from the feature 128 × 128 space to the 1024 × 1024 output space. In contrast, the retrieval-augmented mask leverages the high-resolution nature of real physical samples from the database. This process effectively fills pixel voids and eliminates upsampling noise, markedly improving the IoU and generating spatially continuous skeleton structures and geometric continuity. Extended visualization results of reconstructed samples are provided in fig. S7 and movie S3. In addition, detailed testing protocols and results for flexible arms with different specifications (1.4 mm in diameter, 20 and 40 mm in lengths) are presented in fig. S8.

We further quantified this performance using box plots (Fig. 8B) across the three datasets. Statistical results indicate that the RAG strategy yields a substantial performance improvement across all test scenarios, effectively shifting the IoU distribution toward a higher confidence interval. Specifically, the strategy elevated the average IoU by ~12 to 18% across the datasets (e.g., boosting IoU from 0.691 to 0.871 in the most challenging second dataset), ultimately achieving an average IoU of 0.931 on the comprehensive test set of 10,107 frames. This confirms that the introduction of physical priors effectively corrects the geometric biases inherent in deep networks. Furthermore, addressing the stringent safety requirements of medical interventions, we adapted the confidence gating mechanism from our discrete state confirmation strategy to implement an outlier rejection policy for continuous morphological reconstruction (Fig. 8C). We defined the retrieval match score (i.e., the overlap ratio between the predicted mask and the best-matching database sample) as a confidence metric. When this overlap falls below 50%, the system determines that the current prediction has deviated from the valid physical manifold (indicating a potential hallucination) and actively rejects the frame rather than providing erroneous navigation cues. The threshold setting is designed to balance reconstruction fidelity and navigational continuity and can be flexibly adjusted according to specific clinical safety requirements. Experimental results on the third dataset show that this mechanism successfully identified and filtered out 0.5% of low-confidence data. This conservative strategy ensures that the system provides feedback only when supported by sufficient physical evidence, thereby markedly enhancing the robustness and safety of surgical navigation.

To verify that the proposed system meets the real-time requirements of intraoperative closed-loop control and digital twinning, we have systematically tested and optimized the end-to-end inference latency. Detailed real-time performance analysis, timing data, and visualization overhead evaluation are provided in Supplementary Text and fig. S9.

## DISCUSSION

In summary, we have presented and validated a deep learning–enabled versatile perception method based on a minimalist single-ended MMF system. Distinct from traditional strategies that rely on stacking discrete sensors, our approach shifts the complexity from physical integration to neural decoding. This research not only provides a versatile, scalable, and intervention-compatible shape feedback solution for soft robots but elevates the system from simple quantitative sensing to scenario-adaptive perception. Enabled by distinct neural decoders integrating designs such as confidence gating, geometric encoding, view constraints, and retrieval augmentation, we achieve multidimensional capabilities from a single unified optical interface, spanning discrete state confirmation (point), continuous shape tracking (line), and 3D reconstruction (surface). Empirical validations across three heterogeneous soft robotic scenarios demonstrate that this minimalist physical medium, augmented by data-driven intelligence, suffices to support the complex proprioception required by embodied agents, laying a versatile foundation for closed-loop control in next-generation digital twins.

Although the system demonstrates robust performance across various tasks, its transition from a laboratory prototype to clinical applications presents broad opportunities for optimization. First, at the physical mechanism level, the output speckle patterns are extremely sensitive to external environmental changes such as temperature fluctuations and mechanical vibrations. This instability causes ambient noise to interfere with the target sensing signals, making it a critical bottleneck to isolate true state changes from a drift-prone speckle field for practical applications. To this end, we have implemented an online learning strategy based on KL divergence (fig. S10), demonstrating that the system can effectively suppress such environmental drifts in long-term perturbation scenarios without the need for full offline recalibration. However, when facing cross-hardware deployment or sensing task migration, the integration of online transfer learning offers a practical technical path (*38*, *39*). By extracting and transferring universal physical priors, future systems are expected to overcome the impacts of system drifts and environmental perturbations with minimal data overhead and lightweight network fine-tuning, thereby breaking the rigid dependence of optical systems on specific hardware and scenarios. Ultimately, this adaptive framework is poised to evolve from passive error compensation to active multiphysical sensing. By incorporating mechanisms such as wavelength division multiplexing (*40*, *41*) or mode-selective excitation, environmental perturbations can be inverted into decoupled measurements of temperature fields (*42*) or vibration modes (*43*). Such an evolution would transform the inherent environmental sensitivity of MMF from a calibration challenge into a robust asset for environment-adaptive, multimodal perception.

Beyond physical robustness, the evolution of algorithmic coherence represents the next computational frontier. While our current “divide-and-conquer” strategy ensures precision for distinct tasks, it has not yet established a unified, end-to-end adaptive workflow. The emergence of foundation models and neuromorphic computing offers a transformative pathway to bridge this gap (*44*–*46*). We envision next-generation decoders evolving from discrete task-specific networks into a single, scenario-adaptive perception system. By synthesizing diverse output modalities into a coherent cognitive stream, such a system would mirror biological nervous systems, where comprehensive capabilities, ranging from contact detection and deformation tracking to semantic understanding, emerge organically from continuous optical flow (*47*).

To physicalize this brain-like computational architecture, deep hardware integration offers promising evolutionary paths. On-chip optical neural networks (*48*–*50*), for instance, can use high-speed photon propagation to perform feature extraction directly (*51*), potentially compressing the entire demodulation system to the millimeter scale while providing ultralow latency, low-power support for long-term implantable biological monitoring. Meanwhile, the fusion of flexible photonics will allow deep integration of fibers with flexible circuits. This enables the construction of optoelectronic smart skins, achieving distributed synergy between skeletal proprioception and local tactile feedback.

Ultimately, the synergy between these evolving computational frameworks and integrated hardware empowers the system to transcend the boundaries of unimodal perception. The fiber itself is a natural carrier for imaging (*52*) (as preliminarily demonstrated in fig. S11), implying that a single fiber can integrate perception and vision, simultaneously undertaking instrument navigation and in situ pathological imaging. In addition, using the fiber’s spectral transmission properties, the system can extend to theranostics. It can monitor tissue microenvironments or transmit high-energy lasers for ablation, creating a closed loop of perception (*53*), diagnosis, and therapy. This positions the system as a core hub for next-generation intelligent minimally invasive surgical robots.

In conclusion, this work not only leverages the perception potential of standard fibers through computational reconfiguration but also establishes a versatile, cost-effective strategy for resolving the perceptual mismatch in soft robotics. Ultimately, this closed-loop nervous system, fusing photonic physics with computational intelligence, transcends the boundaries of traditional sensors. It serves as a robust perceptual foundation for next-generation embodied intelligence systems with digital twin capabilities, driving a fundamental shift from passive measurement to autonomous cognition in complex unstructured environments across medical, industrial, and aerospace frontiers.

## MATERIALS AND METHODS

### Experimental design

We constructed a highly integrated single-ended reflective sensing system based on a standard step-index MMF (Nufern, LMA-GDF-30/250-M). The complete optical system structure was illustrated in fig. S1. For illumination, a 1064-nm single-mode laser (Connet, CoSF-D-YB-M-1064-PM-FA) was delivered through a transmission fiber [YOFC, 135/155, 0.22/0.46 numerical aperture (NA)] and injected into the signal MMF via a side coupling module. This side coupling strategy enhances the excitation of high-order modes to enrich the generated speckle fields. Upon reaching the distal end, the light is reflected by the fiber end-face and travels back to the proximal end. This back-reflected speckle field, which encodes bending information from the entire fiber segment, is imaged via an objective lens onto a monochrome complementary metal-oxide semiconductor camera (MER2-301-125U3M, 2048 × 1536 pixels, 8-bit depth).

To quantify the number of modes excited by the side coupling strategy, we performed theoretical calculations based on waveguide theory and the weakly guiding approximation. The sensing fiber used in this work was a standard step-index double-clad fiber (Nufern LMA-GDF-30/250-M) operating at a wavelength of λ = 1064 nm, with a core diameter of 2*a*_core_ = 30 μm with NA_core_ = 0.065, as well as an inner cladding diameter of 2*a*_clad_ = 250 μm with NA_clad_ = 0.46. The total number of guided modes supported by a step-index fiber can be estimated using the formula $M\approx \frac{V^{2}}{2}$, where *V* is the normalized frequency defined by $V=\frac{2\pi a}{\lambda }\mathrm{ΝΑ}$ (*a* is the radius of the core or inner cladding). For core-only light propagation, we obtain *V*_core_ ≈ 5.76, supporting approximately 17 modes. In contrast, side coupling injects light into the inner cladding, resulting in *V*_clad_ ≈ 339.3 and enabling the excitation of approximately 57,560 modes. This calculation confirms that the side coupling strategy enhances the number of excited modes, enriches the information encoding capacity of the speckle field, and lays the foundation for high-sensitivity shape perception.

To process the raw optical signals, we implemented a multistage image preprocessing pipeline. First, we cropped a central region of interest of 768 × 768 pixels from the original image to exclude edge regions with unstable gradients. Subsequently, to reduce computational load while preserving high-frequency features, we applied a 3:1 discrete downsampling strategy (selecting every third pixel). Last, the processed image was resized to 128 × 128 pixels to serve as the input for the neural network.

### Discrete state confirmation

To rigorously quantify the performance boundaries of the proposed anchor-unseen confirmation strategy, we first established a standard verification platform using a high-precision motorized rotation stage (Hengyang LZP-5; absolute accuracy: 0.005°, repeatability: 0.002°). Unlike the complex, coupled deformations inherent in soft robots which lack absolute ground truth, this rigid setup provides precise, decoupled angular labels. This allows us to strictly define anchor (calibrated) and unseen (transition) intervals, establishing a clean baseline for evaluating the model’s selectivity. A 50-mm MMF sensing segment was fixed to the stage, with the bending state defined by the distal tip angle within a ±90° range. We performed periodic angular scanning (9 cycles in total) to collect data across 180 discrete states at 1° intervals. Data from the first 8 cycles (~57,600 speckles) constituted the training and validation sets, while the 7200 unique images from the ninth cycle formed an independent test set. This split simulates the complete workflow from calibration to application, ensuring a robust evaluation of model generalization.

The discrete classifier adopted a lightweight VGG-style network, with a core architecture comprising four convolutional blocks (each sequentially containing a Conv2d layer, Tanh activation function, and MaxPool2d layer), followed by a Dropout layer to suppress overfitting and two fully connected layers to output anchor state classification results. To prevent overfitting on the high-dimensional optical data, we implemented a fivefold cross-validation strategy, training five independent subnetworks on nonoverlapping subsets and integrating their outputs via a soft voting mechanism. Crucially, to quantify prediction certainty, we used the KL divergence between the network’s output probability distribution *p* and a uniform distribution *U*. To ensure that the threshold metric is scale invariant across tasks with different class counts, we normalized the raw KL divergence by the maximum theoretical entropy (ln*N*, where *N* is the number of anchor states)

$$\text{Confidence}=\frac{D_{\text{KL}}\left(p∥U\right)}{\text{ln}N}=\frac{\sum p_{i}\text{ln}\left(p_{i}N\right)}{\text{ln}N}$$ (3)

This normalization maps the confidence score to a standardized [0, 1] interval. Only samples with a normalized confidence exceeding 0.95 are classified as valid anchors, while others are rejected as unseen states.

Transferring this strategy to a soft gripper introduces new challenges: Unlike the distinct boundaries observed in rigid platform experiments, soft robotic motions exhibit ambiguous transition zones between states, which may introduce interference. To address this, we introduced a train unseen mechanism incorporating hard negative mining. Instead of solely evaluating the capability to handle unseen states during the validation phase, we actively incorporated negative samples into the training process. Specifically, we used the PCC of adjacent frames to filter the training data, selecting images corresponding to rapid, chaotic deformations. These images were labeled as “hard negatives” to distinguish true dynamic states from the blurred boundaries of anchors. Subsequently, we used a dual loss function

$$L_{\text{total}}=-\sum_{c=1}^{N}y_{c}\text{log}\left(p_{c}\left(x_{\text{anc}}\right)\right)+\lambda \sum_{c=1}^{N}p_{c}\left(x_{\text{neg}}\right)\text{log}\left(p_{c}\left(x_{\text{neg}}\right)\right)$$ (4)

Here, the first term (cross-entropy) aligns the predictions of anchor samples with ground truth labels; the second term (negative entropy) forces the probability distribution of negative samples toward uniformity, thereby explicitly suppressing false triggers during dynamic transitions. λ denotes the weight factor that balances the contributions of the two loss terms.

### Continuous super-resolution regression strategy

To overcome the noise sensitivity inherent in direct scalar regression, we implemented a structured geometric encoding strategy that transforms the 1D angular regression task into a 2D mask prediction problem. This design leverages the spatial continuity of the mask manifold to regularize the learning process. The regression model uses a U-Net architecture integrated with CBAM. The encoder comprises four downsampling stages, each consisting of two 3 × 3 convolutional layers followed by batch normalization, rectified linear unit (ReLU) activation, and 2 × 2 MaxPooling. A CBAM block is inserted after each stage to adaptively refine features: The channel attention mechanism reweights spectral feature maps, while the spatial attention mechanism localizes the deformation-sensitive regions (i.e., the edges of the fan ribs). The decoder uses 2 × 2 transposed convolutions for upsampling and recovers fine-grained spatial details via skip connections that concatenate low-level features from the encoder. To enforce both pixel-level accuracy and structural completeness, we designed a composite loss function

$$L_{\text{total}}=L_{\text{MSE}}+\lambda L_{\text{IoU}}=\frac{1}{N}\sum {\left(y-y_{\text{pred}}\right)}^{2}+\lambda \left(1-\frac{|y\cap y_{\text{pred}}|}{|y\cup y_{\text{pred}}|}\right)$$ (5)

where *y* and *y*_pred_ represent the ground truth and predicted masks, respectively, and λ was set to 0.5 to balance the two terms. *L*_MSE_ ensures pixel-wise fidelity, while *L*_IoU_ promotes global shape consistency.

Furthermore, to validate the topological universality of this encoding strategy, we tested an alternative “terrace-type” geometric encoding scheme, in which the bending angle is linearly mapped to the vertical position of a horizontal stripe within the 2D mask. This alternative topology yielded performance comparable to the fan-rib design (MAE = 0.012°, detailed results are provided in fig. S6), confirming that the method’s robustness stems from the dimensionality expansion strategy rather than a specific mask geometry.

To validate the generalization capability of the regression strategy under complex multijoint coupling conditions, we integrated the sensing fiber into the index finger of a bionic dexterous hand. The fiber was embedded along the dorsal side, traversing three articulated joints to capture global morphological changes from complete extension to maximum flexion. For the dual-metric evaluation mentioned in Results, the interval hit rate for uncalibrated transition zones is formally defined on the basis of a monotonicity constraint: A prediction *y*_pred_ is considered a valid “hit” if it falls strictly within the angular bounds of its corresponding bounding anchors, i.e., $y_{\text{anchor}}^{\left(i\right)}\leq y_{\text{pred}}\leq y_{\text{anchor}}^{\left(i+1\right)}$. This metric quantifies the logical correctness of the continuous tracking in dynamic intervals where absolute ground truth is unavailable.

### 3D morphological reconstruction and RAG

The dual-view ground truth acquisition system uses two cameras (1024 × 1024 pixels) orthogonally positioned around the sensing workspace. To ensure temporal alignment, these cameras are synchronized with the speckle acquisition camera via hardware trigger signals from a data acquisition (DAQ) module (NI USB 6211). The captured orthogonal images are binarized and processed using epipolar geometry to reconstruct high-precision 3D skeleton ground truth.

The backbone of DVS-Net implements a hierarchical feature extraction structure with four stages (channel dimensions: 32, 64, 128 and 256). Stacked convolutional blocks perform downsampling to extract multiscale features from the speckles. To resolve the geometric ambiguity in reconstructing 3D morphology from single-fiber 2D speckles, we designed a latent cross-view encoder. This module concatenates learnable view tokens (*Q_A_* and *Q_B_*), shared skeleton latent tokens, and the flattened multiscale feature sequences, feeding them into a transformer encoder with eight attention heads and six layers. Through a global self-attention mechanism, the view tokens interact comprehensively with the feature sequences, extracting view-specific geometric information while enforcing consistency between orthogonal views. The decoder uses 2 × 2 transposed convolutions for upsampling, generating coarse probability masks (128 × 128) via Sigmoid activation.

To overcome the resolution bottleneck of end-to-end generation and eliminate geometric hallucinations, we implemented a retrieval-based postprocessing strategy. During the offline phase, we constructed a prior database containing high-res ground truth masks (1024 × 1024) derived from physical experiments. During online inference, the coarse mask predicted by DVS-Net is flattened into a binary vector and projected into a compact 128-dimensional feature space using PCA. This vector serves as a query for a KNN search within the database. The final high-resolution mask is generated by a weighted voting fusion of the retrieved top-K physical samples. As stated in Results, frames with a retrieval overlap score below 0.5 are rejected to ensure safety.

## References

[1] A. Gupta, S. Savarese, S. Ganguli, L. Fei-Fei, Embodied intelligence via learning and evolution. Nat. Commun. 12, 5721 (2021). 34615862 10.1038/s41467-021-25874-z PMC8494941

[2] X. Li, X. Xiao, X. Xiao, Z. Liu, J. Gong, Z. Lin, B. Xue, S. Liu, X. Wu, W. Zhang, D. Wang, R. Zhao, Z. Wang, X. Zhong, Y. Lin, P. Chia, X. He, J. S. Ho, G. W. Ho, W. Ouyang, W. Ding, G. Zhou, C. Laschi, C. Wu, Magnetic field–enhanced vertical integration enables embodied intelligence in untethered soft robots. Sci. Adv. 11, eadv9572 (2025). 40929269 10.1126/sciadv.adv9572 PMC12422188

[3] Y. Kim, G. Parada, S. Liu, X. Zhao, Ferromagnetic soft continuum robots. Sci. Robot. 4, eaax7329 (2019). 33137788 10.1126/scirobotics.aax7329

[4] W. Mu, W. Zhao, B. Li, Y. Liu, R. Yin, A novel piezoelectric-driven and sensing soft robot. Adv. Mater. 10, e01142 (2025).

[5] C. S. X. Ng, M. W. M. Tan, C. Xu, Z. Yang, P. S. Lee, G. Z. Lum, Locomotion of Miniature Soft Robots. Adv. Mater. 33, 2003558 (2020). 10.1002/adma.202003558 33338296

[6] B. Shih, D. Shah, J. Li, T. G. Thuruthel, Y.-L. Park, F. Iida, Z. Bao, R. Kramer-Bottiglio, M. T. Tolley, Electronic skins and machine learning for intelligent soft robots. Sci. Robot. 5, eaaz9239 (2020). 33022628 10.1126/scirobotics.aaz9239

[7] M. Khatib, E. Zhao, S. Wei, J. Park, A. Abramson, E. Bishop, A.-L. Thomas, C.-H. Chen, P. Emengo, C. Xu, R. Hamnett, S. Root, L. Yuan, M. Wurdack, T. Zaluska, Y. Lee, K. Parkatzidis, W. Yu, D. Chakhtoura, Z. Bao, High-density soft bioelectronic fibres for multimodal sensing and stimulation. Nature 645, 656–664 (2025). 40962977 10.1038/s41586-025-09481-2

[8] X. Liu, H. Tang, N. Li, L. He, Y. Tian, B. Hao, J. Xue, C. Yang, J. J. Y. Sung, L. Zhang, J. Zang, Miniature magneto-ultrasonic machines for wireless robotic sensing and manipulation. Sci. Robot. 10, eadu4851 (2025). 40961211 10.1126/scirobotics.adu4851

[9] T. G. Thuruthel, B. Shih, C. Laschi, M. T. Tolley, Soft robot perception using embedded soft sensors and recurrent neural networks. Sci. Robot. 4, eaav1488 (2019). 33137762 10.1126/scirobotics.aav1488

[10] D. Hu, F. Giorgio-Serchi, S. Zhang, Y. Yang, Stretchable e-skin and transformer enable high-resolution morphological reconstruction for soft robots. Nat. Mach. Intell. 5, 261–272 (2023).

[11] K. Kim, J.-H. Hong, K. Bae, K. Lee, D. J. Lee, J. Park, H. Zhang, M. Sang, J. E. Ju, Y. U. Cho, K. Kang, W. Park, S. Jung, J. W. Lee, B. Xu, J. Kim, K. J. Yu, Extremely durable electrical impedance tomography–based soft and ultrathin wearable e-skin for three-dimensional tactile interfaces. Sci. Adv. 10, eadr1099 (2024). 39303034 10.1126/sciadv.adr1099 PMC11414730

[12] P. Wang, Z. Xie, W. Xin, Z. Tang, X. Yang, M. Mohanakrishnan, S. Guo, C. Laschi, Sensing expectation enables simultaneous proprioception and contact detection in an intelligent soft continuum robot. Nat. Commun. 15, 9978 (2024). 39557876 10.1038/s41467-024-54327-6 PMC11574004

[13] T. Yushuang, X. Meng, D. Tao, L. Dongquan, F. Chen, Upper limb motion tracking with the integration of IMU and Kinect. Neurocomputing 159, 207–218 (2015).

[14] T. Kim, S. Lee, T. Hong, G. Shin, T. Kim, Y.-L. Park, Heterogeneous sensing in a multifunctional soft sensor for human-robot interfaces. Sci. Robot. 5, eabc6878 (2020). 33328297 10.1126/scirobotics.abc6878

[15] H. Wang, M. Totaro, L. Beccai, Toward perceptive soft robots: Progress and challenges. Adv. Sci. 5, 1800541 (2018). 10.1002/advs.201800541 PMC6145216 30250796

[16] Z. Lin, Z. Wang, W. Zhao, Y. Xu, X. Wang, T. Zhang, Z. Sun, L. Lin, Z. Peng, Recent advances in perceptive intelligence for soft robotics. Adv. Intell. Syst. 5, 2200329 (2023).

[17] X. Shi, A. Lee, B. Yang, N. Huiming, H. Liu, K. An, H. Liao, H. Kaiyan, J. Wen, X. Luo, L. Zhang, B. Gu, N. Hu, Machine learning assisted electronic/ionic skin recognition of thermal stimuli and mechanical deformation for soft robots. Adv. Sci. 11, e2401123 (2024). 10.1002/advs.202401123 PMC11321626 38864344

[18] H. Zhao, K. O’Brien, S. Li, R. F. Shepherd, Optoelectronically innervated soft prosthetic hand via stretchable optical waveguides. Sci. Robot. 1, eaai7529 (2016). 33157858 10.1126/scirobotics.aai7529

[19] B. Mao, K. Zhou, Y. Xiang, Y. Zhang, Q. Yuan, H. Hao, Y. Chen, H. Liu, X. Wang, X. Wang, J. Qu, A bioinspired robotic finger for multimodal tactile sensing powered by fiber optic sensors. Adv. Funct. Mater. 6, 2400175 (2024).

[20] B. Mao, Y. Xiang, Y. Zhang, Y. Huang, C. Peizheng, G. Cui, J. Qu, A function-structure-integrated optical fingertip with rigid-soft coupling enabling self-decoupled multimodal underwater sensing. Adv. Funct. Mater. 36, e22722 (2026).

[21] Y.-L. Park, S. Elayaperumal, B. Daniel, S. C. Ryu, M. Shin, J. Savall, R. J. Black, B. Moslehi, M. R. Cutkosky, Real-time estimation of 3-D needle shape and deflection for MRI-guided interventions. IEEE/ASME Trans. Mechatron. 15, 906–915 (2010). 26405428 10.1109/TMECH.2010.2080360 PMC4577522

[22] Y. Lu, B. Lu, B. Li, H. Guo, Y.-h. Liu, Robust three-dimensional shape sensing for fexible endoscopic surgery using multi-core FBG sensors. EEE Robot. Autom. Lett. 6, 4835–4842 (2021).

[23] A. Donder, F. R. y. Baena, Dynamic 3-D shape reconstruction for steerable needles with fiber bragg fratings in multicore fibers. IEEE Trans. Robot. 38, 2262–2275 (2022).

[24] E. S. Lamb, Z. Shi, T. Kremp, D. J. DiGiovanni, P. S. Westbrook, Shape sensing endoscope fiber. Optica 11, 1462–1467 (2024).

[25] Y. Meng, R. Sui, W. Liang, H. Zhong, R. Shan, S. Xiao, Y. Kong, C. Fu, Y. Wang, Multicore fiber shape sensing based on optical frequency domain reflectometry parallel measurements. J. Lightwave Technol. 42, 3909–3917 (2024).

[26] H. Cao, T. Čižmár, S. Turtaev, T. Tyc, S. Rotter, Controlling light propagation in multimode fibers for imaging, spectroscopy, and beyond. Adv. Opt. Photonics. 15, 524–612 (2023).

[27] X. Wang, Y. Wang, K. Zhang, K. Althoefer, L. Su, Learning to sense three-dimensional shape deformation of a single multimode fiber. Sci. Rep. 12, 12684 (2022). 35879319 10.1038/s41598-022-15781-8 PMC9314325

[28] L. M. Valentín-Coronado, R. Martínez-Manuel, J. Esquivel-Hernández, M. de los Angeles Martínez-Guerrero, S. LaRochelle, Bending classification from interference signals of a fiber optic sensor using shallow learning and convolutional neural networks. Pattern Recogn. Lett. 186, 354–360 (2024).

[29] C. G. L. Cao, B. Javot, S. Bhattarai, K. Bierig, I. Oreshnikov, V. V. Volchkov, Fiber-optic shape sensing using neural networks operating on multispecklegrams. IEEE Sensors J. 24, 27532–27540 (2024).

[30] X. Wang, R. Wei, Z. Chen, H. Pang, H. Li, Y. Yang, Q. Hua, G. Shen, Bioinspired intelligent soft robotics: From multidisciplinary integration to next-generation intelligence. Adv. Sci. 12, e06296 (2025). 10.1002/advs.202506296 PMC12407382 40583198

[31] L. Wang, Y. Yousi, Z. Liu, J. Tian, Y. Meng, T. Qi, T. He, D. Li, P. Yan, M. Gong, Q. Liu, Q. Xiao, High-speed all-fiber micro-imaging with large depth of field. Laser Photonics Rev. 16, 2100724 (2022).

[32] J. Xu, Y. Mao, Z. Li, Y. Zuo, J. Zhang, B. Yang, W. Xu, N. Liu, Z. J. Deng, W. Chen, K. Xia, C.-W. Qiu, Z. Zhu, H. Jing, K. Liu, Single-cavity loss-enabled nanometrology. Nat. Nanotechnol. 19, 1472–1477 (2024). 39020101 10.1038/s41565-024-01729-8

[33] T. A. Berrueta, A. Pinosky, T. D. Murphey, Maximum diffusion reinforcement learning. Nat. Mach. Intell. 6, 504–514 (2024).

[34] J. Liang, X. Wang, Z. Chen, X. Wei, L. Sun, K. Liu, Z. Shi, T. H. Tao, Z. Zhou, Silk-enabled conformal intraventricular interfaces for minimally invasive neural recordings. Nat. Commun. 16, 9366 (2025). 41130943 10.1038/s41467-025-64397-9 PMC12549829

[35] N. Zhang, P. Zhou, X. Yang, F. Shen, J. Ren, T. Hou, L. Dong, R. Bian, D. Wang, G. Gu, X. Zhu, Biomimetic rigid-soft finger design for highly dexterous and adaptive robotic hands. Sci. Adv. 11, eadu2018 (2025). 40267195 10.1126/sciadv.adu2018 PMC12017302

[36] P. Zhou, J. Lin, W. Zhang, Z. Luo, L. Chen, Pressure-perceptive actuators for tactile soft robots and visual logic devices. Adv. Sci. 9, e2104270 (2022). 10.1002/advs.202104270 PMC8844481 34913616

[37] A. Vaswani, N. Shazeer, N. Parmar, J. Uszkoreit, L. Jones, A. Gomez, L. Kaiser, I. Polosukhin, Attention is all you need. arXiv:1706.03762 [cs.CL] (2017).

[38] F. Jiang, Q. Li, B. Liu, W. Wang, C. Shan, Z. Sun, M. H. Yang, Learning knowledge-based prompts for robust 3D mask presentation attack detection. IEEE Trans. Pattern Anal. Mach. Intell. 48, 1321–1338 (2026). 41052112 10.1109/TPAMI.2025.3618630

[39] C. Ma, A. Li, Y. Du, H. Dong, Y. Yang, Efficient and scalable reinforcement learning for large-scale network control. Nat. Mach. Intell. 6, 1006–1020 (2024).

[40] D. J. Richardson, J. M. Fini, L. E. Nelson, Space-division multiplexing in optical fibres. Nat. Photonics 7, 354–362 (2013).

[41] H. Ren, J. Jang, C. Li, A. Aigner, M. Plidschun, J. Kim, J. Rho, M. A. Schmidt, S. A. Maier, An achromatic metafiber for focusing and imaging across the entire telecommunication range. Nat. Commun. 13, 4183 (2022). 35853875 10.1038/s41467-022-31902-3 PMC9296535

[42] W. Mei, Z. Liu, C. Wang, C. Wu, Y. Liu, P. Liu, X. Xia, X. Xue, X. Han, J. Sun, G. Xiao, H. Tam, J. Albert, Q. Wang, T. Guo, Operando monitoring of thermal runaway in commercial lithium-ion cells via advanced lab-on-fiber technologies. Nat. Commun. 14, 5251 (2023). 37640698 10.1038/s41467-023-40995-3 PMC10462619

[43] H. He, L. Jiang, Y. Pan, A. Yi, X. Zou, W. Pan, A. Willner, X. Fan, Z. He, L. Yan, Integrated sensing and communication in an optical fibre. Light Sci. Appl. 12, 25 (2023). 36650159 10.1038/s41377-022-01067-1 PMC9845349

[44] H. Yu, Z. Huang, S. Lamon, B. Wang, H. Ding, L. Jian, Q. Wang, H. Luan, M. Gu, Q. Zhang, All-optical image transportation through a multimode fibre using a miniaturized diffractive neural network on the distal facet. Nat. Photonics 19, 486–493 (2025).

[45] C. Schuman, S. Kulkarni, M. Parsa, J. Mitchell, P. Date, B. Kay, Opportunities for neuromorphic computing algorithms and applications. Nat. Comput. Sci 2, 10–19 (2022). 38177712 10.1038/s43588-021-00184-y

[46] S.-O. Park, H. Jeong, J. Park, J. Bae, S. Choi, Experimental demonstration of highly reliable dynamic memristor for artificial neuron and neuromorphic computing. Nat. Commun. 13, 2888 (2022). 35660724 10.1038/s41467-022-30539-6 PMC9166790

[47] X. Tu, C. Qian, T. Feng, Y. Zhen, B. Cui, T. Li, L. Tong, L. Zhang, Insect-inspired micro-optical antenna enables ultrasensitive multisensory perception. Sci. Adv. 11, eaec4252 (2025). 41370388 10.1126/sciadv.aec4252 PMC12693968

[48] F. Ashtiani, A. Geers, F. Aflatouni, An on-chip photonic deep neural network for image classification. Nature 606, 501–506 (2022). 35650432 10.1038/s41586-022-04714-0

[49] H. Zhang, M. Gu, X. D. Jiang, J. Thompson, H. Cai, S. Paesani, R. Santagati, A. Laing, Y. Zhang, M. H. Yung, Y. Z. Shi, F. K. Muhammad, G. Q. Lo, X. S. Luo, B. Dong, D. L. Kwong, L. C. Kwek, A. Q. Liu, An optical neural chip for implementing complex-valued neural network. Nat. Commun. 12, 457 (2021). 33469031 10.1038/s41467-020-20719-7 PMC7815828

[50] Z. Gu, Y. Shi, Z. Zhu, Z. Li, M. Zou, C. Yang, Y. Liu, X. Zhang, All-integrated multidimensional optical sensing with a photonic neuromorphic processor. Sci. Adv. 11, eadu7277 (2025). 40446046 10.1126/sciadv.adu7277 PMC12124386

[51] Z. Xue, Z. Tiankuang, Z. Xu, S. Yu, Q. Dai, L. Fang, Fully forward mode training for optical neural networks. Nature 632, 280–286 (2024). 39112621 10.1038/s41586-024-07687-4 PMC11306102

[52] Z. Wen, Z. Dong, Q. Deng, C. Pang, C. F. Kaminski, X. Xu, H. Yan, L. Wang, S. Liu, J. Tang, W. Chen, X. Liu, Q. Yang, Single multimode fibre for in vivo light-field-encoded endoscopic imaging. Nat. Photonics 17, 679–687 (2023).

[53] T. Merk, R. M. Köhler, T. M. Brotons, S. R. Vossberg, V. Peterson, L. F. Lyra, J. Vanhoecke, M. Chikermane, T. S. Binns, N. Li, A. Walton, C. Neudorfer, A. Bush, N. Sisterson, J. Busch, R. Lofredi, J. Habets, J. Huebl, G. Zhu, Z. Yin, B. Zhao, A. Merkl, M. Bajbouj, P. Krause, K. Faust, G.-H. Schneider, A. Horn, J. Zhang, A. A. Kühn, R. M. Richardson, W.-J. Neumann, Invasive neurophysiology and whole brain connectomics for neural decoding in patients with brain implants. Nat. Biomed. Eng., doi: 10.1038/s41551-025-01467-9 (2025). 10.1038/s41551-025-01467-9 PMC13190260 40993190

