# Supplementary material for: Deep learning–enabled versatile shape perception for soft robots via single-ended multimode fiber
Source: Sci Adv. 2026 Jun 12;12(24):eaef6263. doi: 10.1126/sciadv.aef6263 (PMC13262633; doi:10.1126/sciadv.aef6263)
Supplement: Supplementary file 1 — Supplementary Text Figs. S1 to S11 Legends for movies S1 to S3 [file sciadv.aef6263_sm.pdf]

Science Advances
AAAS

Supplementary Materials for

Deep learning–enabled versatile shape perception for soft robots via single-ended multimode fiber

Zhaofan He *et al.*

Corresponding author: Qiang Liu, qiangliu@tsinghua.edu.cn; Qirong Xiao, xiaoqirong@mail.tsinghua.edu.cn

Sci. Adv. **12**, eaef6263 (2026)
DOI: 10.1126/sciadv.aef6263

The PDF file includes:

Supplementary Text
Figs. S1 to S11
Legends for movies S1 to S3

Other Supplementary Material for this manuscript includes the following:

Movies S1 to S3

Supplementary Text

1. Physics of speckle-based shape encoding via mode coupling

In MMF sensing, geometric deformations (such as bending and torsion) of the distal sensing segment modulate the energy coupling between modes by altering the fiber's refractive index profile via the elasto-optic effect. This physical process is governed by the transmission matrix theory. Assume the length of the distal sensing segment is L, and the fiber supports N propagating modes. The complex amplitudes of these modes at the input end form a column vector:

$$\mathrm{c}(0)=\begin{pmatrix} c_1(0) \\ c_2(0) \\ c_3(0) \\ \vdots \\ c5(0) \end{pmatrix} \qquad (S1)$$

Where $c_\mu(0)$ represents the complex amplitude of the μ-th mode at the input end. This input mode amplitude vector serves as the crucial link connecting the actual optical field with the theoretical model. Its physical essence lies in the projection of the input optical field $E_{in}(r,\phi)$ onto the respective modes, meaning the input field can be decomposed into a linear superposition of these modes:

$$E_{in}(r,\varphi)=\sum\nolimits_{\mu=1}^{N} c_\mu(0)E_\mu^{(t)}(r,\varphi) \qquad (S2)$$

where $E_\mu^{(t)}(r,\phi)$ denotes the transverse electric field distribution of the μth mode. Through the orthogonality condition:

$$\frac{\beta_\mu}{2\omega_0}\int_0^\infty\int_0^{2\pi} \mathrm{r}E_\mu^{(t)*}E_v^{(t)}d\varphi dr=\delta_{\mu,v} \qquad (S3)$$

$c_\mu(0)$ can be uniquely determined:

$$\mathrm{c}_\mu(0)=\frac{\beta_\mu}{2\omega_0}\int_0^\infty\int_0^{2\pi} rE_{in}(r,\varphi)E_\mu^{(t)*}(r,\varphi)d\varphi dr \qquad (S4)$$

Where $c(z)$ denotes the complex amplitude of each of the N modes propagating at position z along the fiber axis, the dependence of c on z arises solely from mode coupling and is governed by the following equation:

$$\frac{\mathrm{d}c}{\mathrm{d}z} = -j(\beta + K)c \tag{S5}$$

Where $\beta = diag(\beta_1, \beta_2, ..., \beta_N)$, the diagonal matrix of propagation constants β_i for the ideal model, and the $N \times N$ matrix K represents the coupling (or equivalent coupling rate) per unit length. Its elements can be evaluated through integration:

$$K_{\mu,v} = \frac{\omega}{j4P} \int_0^{\infty} \int_0^{2\pi} r E_{\mu}^{*} \Delta\varepsilon E_v d\varphi dr = K_{v,\mu}^{*} \tag{S6}$$

$\Delta\varepsilon$ is a 3×3 perturbation matrix added to the dielectric tensor of the ideal fiber, while the 3D vectors E_μ and E_v represent the complete electric field of modes μ and v, including both transverse and longitudinal components. To quantify the coupling matrix $E_{\mu,v}$, the physical origin of the dielectric perturbation $\Delta\varepsilon$ must be explicitly defined. When the optical fiber is subjected to external physical actions (such as bending, torsion, or compression), complex stress distributions are generated internally. According to the elasto-optic effect, these mechanical stress tensors σ_{ij} directly induce anisotropic perturbations $\Delta\varepsilon$ in the fiber's dielectric tensor:

$$\Delta\varepsilon_{\mathrm{ij}}(r, \varphi, z) = -\varepsilon_{ii}\varepsilon_{jj} \sum_{k,l} p_{ijkl} S_{kl}(r, \varphi, z) \tag{S7}$$

where S_{kl} represents the strain components determined by macroscopic deformations (e.g., curvature κ, torsion rate τ), and p_{ijkl} is the photoelastic tensor. This implies that any macroscopic geometric morphological change—whether tension/compression induced by bending or shear induced by torsion—translates microscopically into a 3D perturbation field of the refractive index distribution $\Delta n(r, \phi, z)$.

We first consider the fundamental case of bending. When the end-sensing segment undergoes only bending perturbation along the x-z plane (curvature κ, corresponding to a bending angle of κL), the internal stress distribution comprises longitudinal stress σ_z and transverse stress σ_x. According to the elasto-optic effect, these stresses are converted into dielectric tensor perturbations via the elastoptic coefficient. Bending induces tensile stress in the outer layer and compressive stress in the inner layer, yielding stresses $\sigma_z = \kappa E_r \cos\varphi$ related to

the radial coordinate r and azimuth φ, where κ is the curvature and E_r is the Young's modulus of silica. The corresponding dielectric tensor perturbation is

$$\Delta\varepsilon_{\text{bending}\_z} = -\kappa n^4 r\cos\varphi\begin{pmatrix} q_1 & 0 & 0 \\ 0 & q_1 & 0 \\ 0 & 0 & q_2 \end{pmatrix} \tag{S8}$$

where $q_1=(1-v)p_{12}-vp_{11}$, $q_2=p_{11}-2vp_{12}$, ν is the Poisson's ratio of silica, and p_{11} and p_{12} are the elastoptic coefficient. Since $q_2 \ll q_1$, the perturbation primarily affects the transverse (x and y directions) dielectric constants, with minimal longitudinal (z-direction) variation. The transverse compressive stress induced by bending is approximately $\sigma_x \approx -\frac{1}{2}\kappa^2 a^2 E$, corresponding to the following dielectric tensor perturbation:

$$\Delta\varepsilon_{\text{bending}\_x} = \frac{1}{2}\kappa^2 a^2 n^4\begin{pmatrix} q_2 & 0 & 0 \\ 0 & q_1 & 0 \\ 0 & 0 & q_1 \end{pmatrix} \tag{S9}$$

The asymmetry of the matrix ($q_2 \neq q_1$) results in coupling between the transverse and longitudinal components. When an optical fiber undergoes torsion around its axis, shear stress induces an asymmetric dielectric tensor perturbation through the elasto-optic effect:

$$\Delta\varepsilon_{\text{torsion}} = \mathrm{g}\,\tau\varepsilon_0 n_{av} r\begin{pmatrix} 0 & 0 & -\sin\phi \\ 0 & 0 & \cos\phi \\ -\sin\phi & \cos\phi & 0 \end{pmatrix} \tag{S10}$$

Where τ is the torsion rate per unit length, $g \approx 0.15$ is the elasticity coefficient, and n_{av} is the average refractive index of the fiber. The off-diagonal elements of the matrix (xz, yz, etc.) indicate that the distortion perturbation primarily couples the transverse and longitudinal electric field components.

Combining the derivations above, in practical soft robotic scenarios, the sensing fiber acts as a continuum that simultaneously undergoes bending and torsion. The total dielectric perturbation is effectively the superposition of the gradient-induced $\Delta\varepsilon_{\text{benging}}$ and the shear-induced $\Delta\varepsilon_{\text{torsion}}$. From the perspective of the transmission matrix, these two deformations play complementary roles in signal encoding: Bending primarily introduces a transverse refractive

index gradient, modifying the diagonal elements of the coupling matrix (inducing propagation constant mismatch and phase shifts). Torsion, via the elasto-optic shear effect, significantly populates the off-diagonal elements, triggering strong energy exchange (mode mixing) between transverse and longitudinal components. Consequently, the evolution of the mode amplitudes follows the integral solution of the coupled-mode equation:

$$\mathrm{c}\left(L\right)=e^{-j(\beta+K_{benging}+K_{torsion})z}c(0) \tag{S11}$$

The single-ended reflective architecture adopted in this study further enhances the deformation-induced phase accumulation effect. In this architecture, after being injected into the fiber via side-coupling, the illumination light propagates forward to the distal sensing segment, is reflected by the fiber end-face, and then propagates back along the original path to the proximal end. Throughout the process, the light completes two round-trip transmissions within the sensing segment, extending the effective sensing optical path from *L* (in the traditional transmissive architecture) to 2*L*. From the perspective of phase evolution, the deformation-induced change in the mode propagation constant $\Delta\beta(\varepsilon(z))$ (where $\varepsilon(z)$ is the axial strain distribution of the fiber) accumulates continuously along the optical path. The total phase change of the reflective architecture is expressed as:

$$\Delta\varphi_{\mathrm{reflect}}=\oint_0^{2L}\Delta\beta(\varepsilon(z))dz=2\oint_0^{L}\Delta\beta(\varepsilon(z))dz=2\Delta\varphi_{transmit} \tag{S12}$$

where $\Delta\phi_{transmit}$ is the phase change of the traditional transmissive architecture. This equation indicates that the reflective structure doubles the deformation-induced phase accumulation, thereby amplifying the inter-mode interference effect. As a result, the speckle field exhibits more distinct characteristic changes under the same deformation. This effect synergizes with the high-order mode excitation enabled by side-coupling, collectively improving the sensing sensitivity of the system.

This formulation reveals that the output speckle field is the result of a highly nonlinear and high-dimensional integral transformation of the fiber's geometric morphology. While this physical complexity renders analytical inversion (reconstructing shape from intensity alone) mathematically ill-posed and computationally intractable using traditional methods, it creates a unique, high-entropy optical "fingerprint" for every morphological state. This physical reality justifies our adoption of deep learning: the neural networks effectively learn the inverse mapping

of this complex multi-physics coupling process, decoding the rich feature stream jointly encoded by bending gradients and torsion shears to achieve versatile shape perception.

In addition, it is necessary to specify that the aforementioned physical derivation of the principles of mode coupling and linear superposition is primarily to verify the unique mapping relationship between fiber deformation and speckle patterns, establish the reliability of speckles as carriers of deformation information, and provide a theoretical foundation for the feature learning of the end-to-end network, rather than directly guiding the design of the network structure.

2. Definition of evaluation metrics and neural network structure

This section serves to supplement the definitions of evaluation metrics not exhaustively detailed in the main text and Methods, and provides a comprehensive introduction to the three neural decoder architectures developed for heterogeneous tasks in this study (detailed network topology can be found in Fig. S2).

Regarding the discrete state confirmation task, we define Anchor Classification Accuracy (ACC_{anchor}) as the core metric for evaluating global model performance. It is primarily used to evaluate the model's ability to correctly identify predefined anchor states, while also accounting for its effectiveness in distinguishing unseen states. Its calculation formula is as follows:

$$\mathrm{ACC}_{anchor} = \frac{TP_{anchor} + TN_{unseen}}{N_{total}} \tag{S13}$$

Here, TP_{anchor} represents the number of samples that are actual anchor states and are correctly predicted as anchors, TN_{unseen} represents the number of samples that are actual unseen states and are correctly predicted as the unseen state, and N_{total} is the total number of anchor and unseen samples in the test set. To further quantify safety during dynamic transitions, we introduce Recall_{unseen} and $\mathrm{Precision}_{unseen}$. Specifically, the Recall_{unseen} represents the proportion of samples that are truly unseen states which are correctly identified as belonging to the unseen state. The calculation formula is as follows:

$$\mathrm{Recall}_{\mathrm{unseen}} = \frac{TN_{unseen}}{TN_{unseen} + FP_{anchor}} \tag{S14}$$

Here, FP_{anchor} represents the number of samples that are actually unseen states but are incorrectly predicted as anchors, while the meanings of the remaining variables are consistent with those in the ACC_{anchor} metric. Complementing this metric, the $Precision_{unseen}$ is used to evaluate the system's ability to avoid false alarms for unseen states, defined as the proportion of samples predicted as the unseen state that are truly unseen states:

$$\text{Precision}_{unseen} = \frac{TN_{unseen}}{TN_{unseen} + FN_{unseen}} \tag{S15}$$

where FN_{unseen} denotes the number of samples that are actually anchor states but are mistakenly predicted as the unseen state.

In the continuous interpolation tracking task, the Mean Absolute Error (MAE) serves as the core metric for evaluating the average deviation of predictions. It measures the mean absolute deviation between the predicted angles and the ground truth angles, calculated as follows:

$$\text{MAE} = \frac{1}{N}\sum_{i=1}^{N}\left|y_{ture,i} - y_{pred,i}\right| \tag{S16}$$

In the formula, N denotes the number of continuous interpolation test samples, $y_{ture,i}$ represents the true bending angle of the i-th sample, and $y_{pred,i}$ is its corresponding predicted angle. A smaller MAE value indicates more consistent and uniformly low deviations in the continuous interpolation process. The Root Mean Square Error (RMSE) is employed as a supplementary metric to assess the dispersion of errors in continuous interpolation. Calculated as the square root of the average of squared prediction errors, it is more sensitive to larger deviations. This sensitivity allows it to reflect the stability of interpolation under extreme conditions. The calculation formula is as follows:

$$\text{RMSE} = \sqrt{\frac{1}{\text{N}}\sum_{i=1}^{N}(y_{ture,i} - y_{pred,i})^2} \tag{S17}$$

The variables in the formula maintain the same definitions as those in the MAE. The Coefficient of Determination is used to quantify the degree of linear correlation between predicted and true values in the continuous interpolation task, reflecting the model's effectiveness in fitting the continuous bending behavior. It is calculated as follows:

$$R^2 = 1 - \frac{\sum_{i=1}^{N}(y_{ture,i} - y_{pred,i})^2}{\sum_{i-1}^{N}(y_{ture,i} - \overline{y}_{ture})^2} \tag{S18}$$

In the formula, $\overline{y}_{ture}$ represents the mean of the true angles. A value of R^2 closer to 1 indicates a better fit.

The Inter over Union (IoU) serves as a key metric for evaluating mask reconstruction accuracy in the dual-view 3D reconstruction task. It quantifies the degree of overlap between the flexible structure mask predicted by the network and the ground truth mask (obtained through HSV color space segmentation and morphological operations). The calculation formula is as follows:

$$\mathrm{IoU} = \frac{Area(Mask_{pred} \cap Mask_{gt})}{Area(Mask_{pred} \cup Mask_{gt})} \tag{S19}$$

Here, $\mathrm{Area}(Mask_{pred} \cap Mask_{gt})$ denotes the intersection area between the predicted mask and the ground truth mask, while $\mathrm{Area}(Mask_{pred} \cup Mask_{gt})$ represents their union area.

Regarding the neural network architectures, the system employs three distinct decoders tailored to heterogeneous tasks, with their specific layer configurations shown in Fig. S2. The discrete state confirmation utilizes a lightweight VGG-based classifier (Fig. S2A) incorporating a confidence gating mechanism to map high-dimensional speckles to anchor states. The continuous shape tracking employs a UNet-based regression network (Fig. S2B) integrated with Convolutional Block Attention Modules (CBAM), transforming 1D angular regression into structured 2D geometric mask prediction to achieve sub-pixel resolution. The 3D morphological reconstruction relies on the DualViewSynergyNet (DVS-Net, Fig. S2C), which features a Latent Cross-View Transformer to enforce geometric consistency between orthogonal views and is further enhanced by a Retrieval-Augmented Generation (RAG) strategy to ensure high-fidelity physical reconstruction.

3. Dataset Construction and Intrinsic Resolution Verification

To construct a high-fidelity training dataset and verify the system's intrinsic resolution limit, we designed a periodic angular scanning protocol as illustrated in Fig. S3A. Utilizing a high-precision motorized rotation stage, a 50-mm MMF sensing segment was driven to perform

periodic scanning within a ±90° range, with the step size strictly controlled at 1°. A total of 9 complete cycles were collected; data from the first 8 cycles constituted the full-state space training set, while the 7,200 samples from the 9th cycle served as an independent test set. This data partitioning strategy simulates the complete engineering workflow from offline calibration to online application. Notably, this full-state dataset encompasses 180 discrete states and serves as the fundamental data pool for the varying Anchor-to-Unseen Ratio (AUR) experiments described in the main text. The anchor and unseen states mentioned in the main text were all sampled and partitioned from this comprehensive dataset according to specific experimental requirements (e.g., AUR=1:9).

The preprocessing method is detailed in Fig. S3B. First, a boundary detection algorithm was applied to crop the effective central speckle region of pixels from the original image. Considering the speckle granularity and balancing feature preservation with computational efficiency, we implemented a 3:1 discrete downsampling strategy, subsequently resizing the image to pixels. This processing significantly reduced data dimensionality while maximally preserving the high-frequency spatial features within the speckle field that encode fiber morphology, thereby preventing the information loss caused by smoothing in traditional interpolation scaling.

Before implementing the confidence gating strategy described in the main text, we first conducted a 180-class full-angle classification experiment based on the aforementioned full-state dataset to verify the sensing system's intrinsic physical resolution. We adopted a 5-fold Cross-Validation strategy, partitioning the training set into five distribution-consistent subsets for sequential validation to eliminate data distribution bias. Fig. S3C demonstrates the baseline classification performance across the full ±90° range (180 states). The confusion matrix shows that the model maintains extremely high classification accuracy even at 1° intervals, with only minor confusion existing between very few adjacent categories. The loss function curves in Fig. S3D display the synchronous convergence of training and validation losses, indicating no overfitting. This baseline verification is of significant importance: it proves that the fiber speckle field physically possesses the capability to resolve minute deformations of 1°. This provides a solid physical foundation for distinguishing anchors from transition states under sparse calibration (e.g., 10° intervals) as described in the main text—specifically, the system is able to

reject intermediate states via confidence scores because these intermediate states are indeed significantly separable from anchors in the feature space.

4. <u>Real-time inference latency evaluation and closed-loop navigation performance</u>

The practical usability of the closed-loop control and digital twin solution during surgery fundamentally depends on whether the cumulative latency of end-to-end online inference is sufficiently low to support high-frequency visual feedback and avoid perceptible visual lag for surgeons.

To this end, we conducted refined decomposition and timing statistics of the inference pipeline for single-frame samples in the context of practical engineering deployment, and defined the online inference latency as:

$$t_{deploy} = t_{data} + t_{\mathrm{mod}el} + t_{knn} + t_{vote} + t_{consistency} \quad \text{(S20)}$$

where t_{data} is the time consumption of input tensor preprocessing and necessary data transmission, t_{model} is the time consumption of dual-view UNet forward propagation to generate 128×128 probability maps, t_{knn} covers the full-process time consumption of constructing retrieval features (downsampling/flattening) from network outputs, PCA dimensionality reduction (to 128 dimensions), and KNN retrieval on approximately 16k sample database; t_{vote} is the time consumption of decompression and majority voting on the bit-compressed high-resolution database resident in GPU to generate 1024×1024 RAG masks; $t_{consistency}$ is the time consumption of consistency IoU verification and joint rejection/trust strategy decision-making, with the final online inference results outputted. It should be clearly stated that the visualization module is an on-demand engineering component in practical clinical systems, and can avoid blocking the main inference pipeline through strategies such as asynchronous display, frame-skipping rendering, or rendering only the region of interest (ROI). Therefore, the visualization latency is not included in the core latency calculation in the above online inference timing statistics, but only quantified and estimated as an independent system overhead in the subsequent part.

We completed the timing benchmark test of the end-to-end online inference pipeline based on the RTX 4090 (24GB) computing platform, and carried out targeted optimization of system configuration accordingly. In the current deployment framework, core computing links such as dual-view U-Net forward propagation, decompression and majority voting of high-resolution

RAG masks, and consistency IoU verification and strategy decision-making are all deployed on the GPU side; PCA feature transformation and KNN retrieval are currently executed on the CPU side, but their input is low-resolution retrieval features output by the network, so the scale of data interaction between CPU and GPU is strictly controlled, effectively avoiding frequent transmission of high-resolution data in the main inference pipeline. As shown in the end-to-end deployment latency distribution comparison in Fig. S9A and the latency breakdown results in Fig. S9B, the baseline version adopts the configuration of KNN neighbor number=5 and retrieval feature resolution of 128×128, with a measured average online deployment latency of 37.7 ms, corresponding to a visual feedback frequency of approximately 26.6 Hz. To meet the demand for >30 Hz high-frequency visual feedback in closed-loop navigation and reduce the risk of visual lag, we optimized the core parameters: adjusting the KNN neighbor number to 3 and reducing the retrieval feature resolution to 96×96. After optimization, the time consumption of the KNN retrieval link was significantly reduced, the end-to-end deployment latency decreased to 21.6 ms (a latency reduction of 42.7%), and the corresponding visual feedback frequency increased to 46.2 Hz; meanwhile, the segmentation performance remained basically stable (Fig. S9C). The average IoU of the baseline version for View A and View B was 0.884 and 0.903, respectively, and the average IoU of the optimized version for View A and View B was 0.888 and 0.899, respectively, with no significant decrease. .

We quantitatively estimated the engineering overhead of the visualization link: when a lightweight display strategy is adopted in the closed-loop system (e.g., simple color overlay on the 1024×1024 predicted mask, display/transmission completed through asynchronous threads, without frame-by-frame storage and complex drawing operations), the visualization link only introduces an additional millisecond-level time consumption (denoted as t_{vis}), and the actual feedback frequency of the system can be approximately expressed as 1/(mean(t_{deploy})+t_{vis}). Taking mean(t_{deploy})=21.6 ms of the optimized version as an example, even if a conservative value of t_{vis}≈2~5 ms ms is taken, the overall feedback frequency of the system can still reach approximately 37 Hz to 42 Hz, both significantly higher than the 30 Hz threshold; under the optimized configuration of asynchronous rendering and frame-skipping display, the feedback frequency can be further close to the 46.2 Hz pure inference upper limit shown in Fig.S9. It should be supplemented that if high-time-consuming operations such as frame-by-frame encoding and storage, and complex drawing based on Matplotlib are incorporated into the main

inference pipeline, the visualization latency will rise to tens of milliseconds and significantly reduce the frame rate. Therefore, such operations are more suitable for offline data recording or frame-skipping quality inspection, rather than the real-time display link for intraoperative closed-loop navigation.

In addition to the existing parameter optimization, the framework still has profound optimization potential in terms of engineering deployment. The current inference pipeline is executed based on the native PyTorch environment; in the future, TensorRT engine can be introduced to achieve operator fusion and FP16 quantization, which is expected to further reduce the U-Net forward inference time consumption (currently about 6.22 ms). For the retrieval link of the 16k sample database, although linear search has achieved millisecond-level response at this stage, professional vector database acceleration libraries such as FAISS can be integrated in the follow-up, and GPU indexing can be used to keep the retrieval latency at a constant level when the sample size expands. Combined with the multi-stream parallel processing characteristics of RTX 4090, the system can pipeline the data preprocessing and model inference processes, ensuring that the digital twin model can provide decision support for surgeons with high physical fidelity and negligible latency in complex dynamic surgical environments.

5. KL-divergence-driven adaptive learning strategy for long-term environmental robustness ehancement

In the research presented in the main text, we proposed a deep learning-enabled versatile shape perception method for soft robots based on a single-ended MMF, which achieves multi-modal perception capabilities including discrete state confirmation, continuous shape tracking, and 3D morphological reconstruction. However, the speckle field of the MMF has inherent cross-sensitivity to external environmental perturbations such as ambient temperature fluctuations and mechanical micro-vibrations. During long-term operation, environmental perturbations will cause non-targeted changes in the mode coupling characteristics within the fiber, leading to continuous drift of the speckle field distribution. This will ultimately result in distribution mismatch between the training data and inference data of the offline trained neural network, triggering continuous degradation of perception performance. This issue is the core challenge that must be addressed for the translation of this technology from controlled laboratory environments to uncontrolled clinical scenarios. To quantify the drift law of the speckle field

distribution caused by environmental perturbations, verify the performance degradation characteristics of the system in uncontrolled environments, and propose an effective performance recovery and robustness enhancement strategy, we carried out systematic long-term environmental perturbation monitoring experiments, and constructed a KL-divergence-based confidence quantification mechanism and a dual-network test-time adaptation learning algorithm. The relevant experimental results and algorithm framework are detailed in Fig. S10 of this supplementary material.

We first designed a long-term speckle acquisition experiment under a static bending state, as shown in Fig. S10A. The MMF was fixed in a constant bending state, and 14,400 speckle images were continuously acquired under uncontrolled conditions with no active deformation, but with an ambient temperature fluctuation of approximately 3 °C (22 °C - 25 °C) accompan ied by micro-vibrations from laboratory equipment. We quantified the drift characteristics of the speckle distribution caused by environmental perturbations using Structural Similarity (SSIM). The experimental results show that the minimum SSIM value is 0.051, the maximum value is 1.000, and the mean value is 0.307. The similarity between the speckle images and the initial state exhibits significant fluctuations and continuous attenuation over time, which directly confirms that environmental perturbations will cause obvious drift in the speckle distribution.

To fundamentally solve the model performance degradation caused by long-term environmental perturbations, based on the above confidence quantification mechanism, we proposed a confidence-aware ensemble learning-based test-time adaptation algorithm, and constructed a dual-network architecture consisting of a Long-term NN and a Short-term NN. Without changing the core knowledge of offline training and introducing additional calibration data, this algorithm realizes dynamic adaptation of the model to environmental perturbations and performance recovery. The dual-network architecture consists of two VGG-type classification networks with exactly the same structure, which is identical to the backbone network used in the discrete state confirmation task in the main text, ensuring complete compatibility between the algorithm and the original perception system. Among them, the Long-term NN is fully trained with offline calibration data, and its network parameters remain fixed during the inference process. Its core function is to retain the core knowledge of the mapping between bending states and speckle distributions learned during the offline training phase, avoid catastrophic forgetting caused by continuous incremental updates, and provide a stable feature benchmark and

knowledge anchor for the entire system; the Short-term NN takes the parameters of the Long-term NN as the initial value, and realizes incremental learning of the current speckle distribution through pseudo labels during the inference process, to adapt to the changes in speckle distribution caused by environmental perturbations in real time.

At the same time, we adopt a mini-batch gradient descent method for parameter update, which avoids severe parameter fluctuations caused by a single update while ensuring update efficiency, and ensures the stability of the model's perception performance. In the confidence-aware selective output module, we fuse the output results of the Long-term NN and the Short-term NN, compare the confidence of the output results of the two networks, and select the result with higher confidence as the final output of the system, so as to balance the knowledge retention ability and environmental adaptability of the model. When the amplitude of environmental perturbation is small and the speckle distribution has no obvious drift, the output confidence of the Long-term NN is higher, and the system outputs results based on the core knowledge of offline training to ensure the stability of the perception results; when the amplitude of environmental perturbation is large and the speckle distribution has obvious drift, the Short-term NN adapts to the current distribution characteristics through incremental learning, its output confidence is higher, and the system takes the adaptively updated result as the output to ensure the accuracy of the perception results, finally achieving a balance between stability and adaptability.

To further solve the problem of performance degradation under long-term environmental perturbations, we proposed a confidence-aware ensemble learning-based test-time adaptation algorithm, as shown in Fig. S10B, and constructed a dual-network architecture consisting of a Static Net and a Dynamic Net. Among them, the Static Net retains the core knowledge from the offline training phase to avoid catastrophic forgetting caused by continuous incremental updates, while the Dynamic Net takes the parameters of the Static Net as the initial value and realizes incremental learning of the current speckle distribution through pseudo labels. The algorithm takes the maximum value of the probability vectors output by the dual networks as the confidence metric, screens high-confidence data to generate pseudo labels for parameter update of the Dynamic Net, and filters low-quality data with confidence below the threshold to ensure the effectiveness of parameter update. To verify the practical effect of this strategy, we conducted a 12.7-hour ultra-long-term test on the real-time MMF bending state sensing system

(as shown in Fig. S10C). The control group Static Net was trained only with initial offline data, without any parameter update or adaptive optimization throughout the test. Under continuous environmental perturbations, its classification accuracy showed a continuous and significant downward trend, dropping from the initial 100% to below 30% in the 12.7-hour test, with a global average accuracy of only 63.80%, which directly verifies that long-term environmental perturbations will cause severe performance degradation without an effective correction strategy. In contrast, the CaSSENet integrated with our proposed adaptive learning strategy maintained a classification accuracy of over 96.88% consistently under the exact same uncontrolled environment and test duration, with a global average accuracy of 99.89% and a maximum accuracy of 100.00%. Only minimal instantaneous fluctuations occurred during severe environmental perturbations, and the accuracy quickly recovered to a high level close to 100%, which fully proves that this algorithm strategy can effectively offset the negative impact caused by environmental perturbations and enable the system to achieve long-term and highly stable perception in uncontrolled environments. This confidence-based adaptive learning framework can be seamlessly transferred to the continuous shape tracking and 3D morphological reconstruction tasks proposed in this paper. By using evaluation metrics such as the predicted mask area as the basis for confidence calculation, the same pseudo label-based incremental learning and confidence screening mechanism can be applied to improve the robustness to environmental perturbations across all perception modes.

It should be specially noted that this confidence-based adaptive learning framework has excellent universality and can be seamlessly transferred to the continuous shape tracking and 3D morphological reconstruction tasks proposed in this paper. In the continuous shape tracking task, the confidence basis can be constructed through evaluation metrics such as the Intersection over Union (IoU) between the predicted mask and the real morphology, and the error distribution of the predicted angle; in the 3D morphological reconstruction task, the consistency of the dual-view predicted masks, retrieval matching score and other indicators can be used as the criteria for confidence judgment. Using the same pseudo label-based incremental learning and confidence screening mechanism, the robustness to environmental perturbations under all perception modes can be improved, which provides complete technical support for the long-term stable operation of the system in complex clinical scenarios.

Fig. S1.

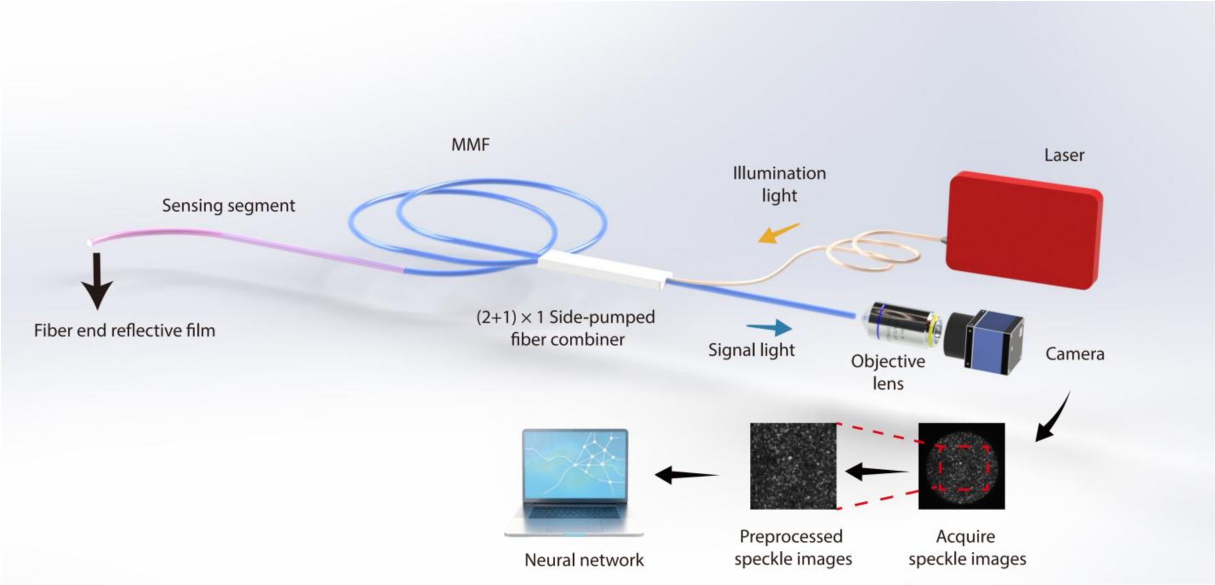

Schematic of the experimental optical setup.

Fig. S2.

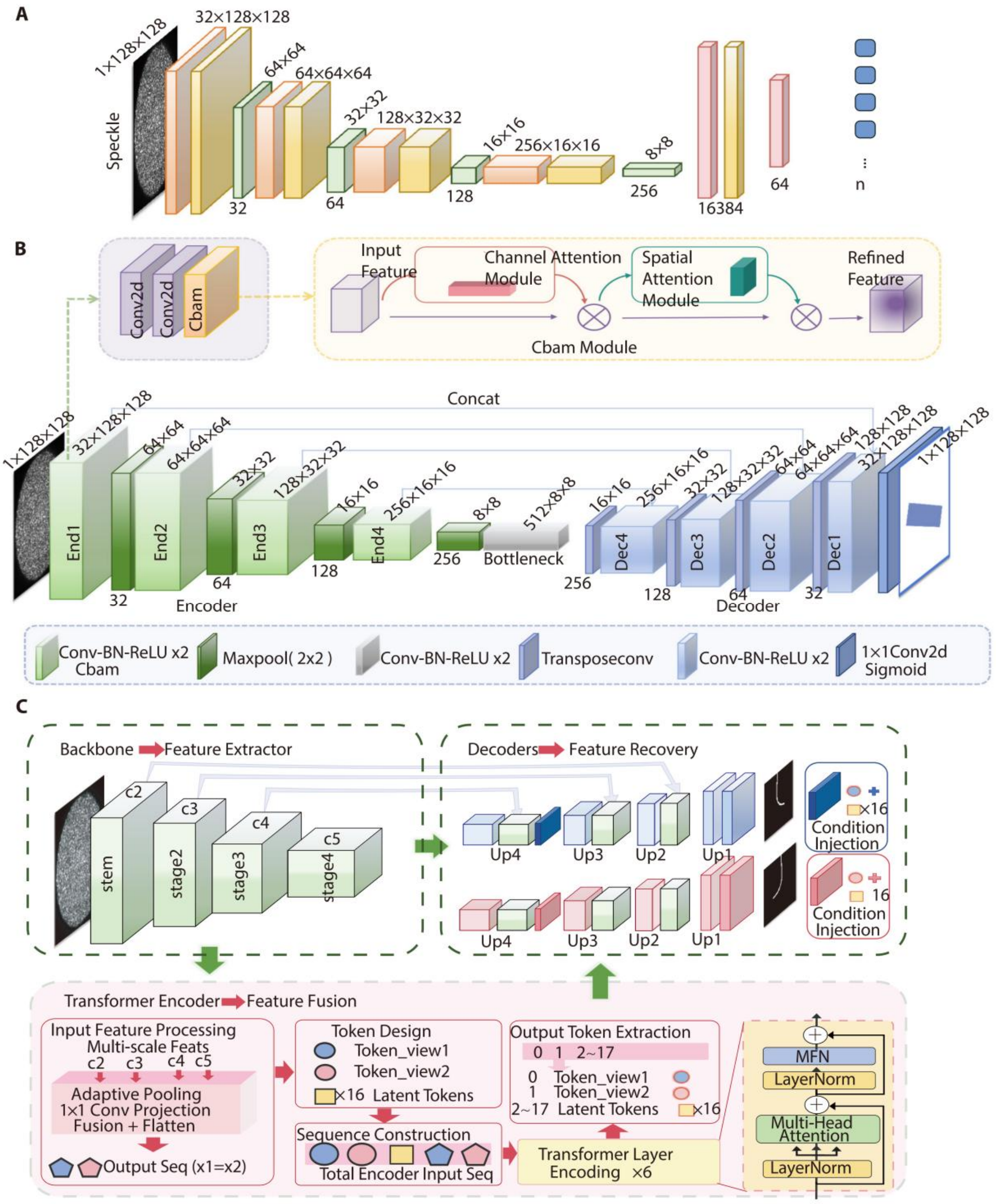

Neural network architectures. (A) The VGG-based network. **(B)** The CBAM-integrated UNet. **(C)** The DualViewSynergyNet (DVS-Net).

Fig. S3.

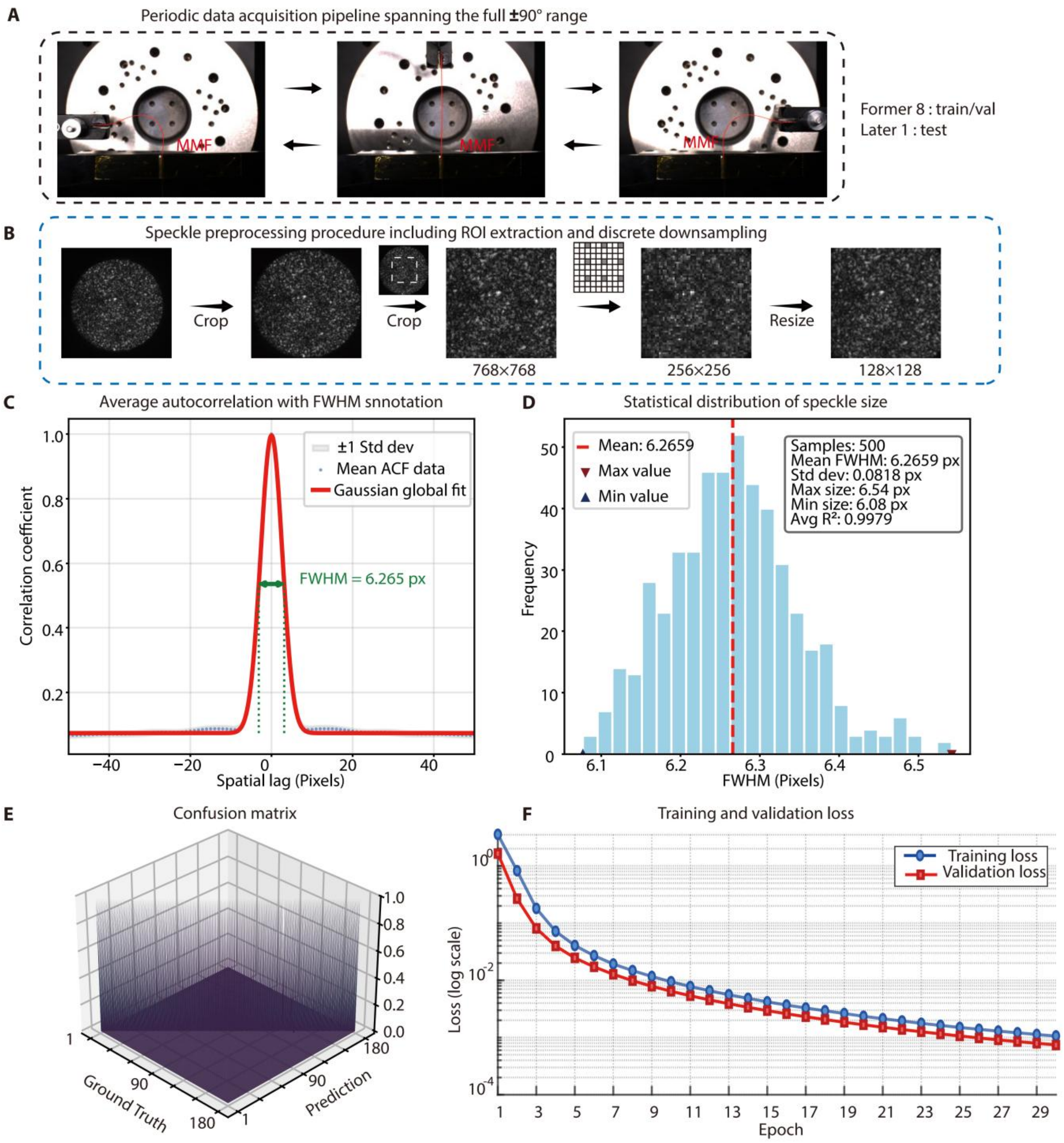

Dataset construction and intrinsic resolution verification. (A) Periodic data acquisition pipeline spanning the full ±90° range. **(B)** Speckle preprocessing procedure including ROI extraction and discrete downsampling. **(C)** Average autocorrelation function (ACF) of speckle patterns with Gaussian fitting, where the full width at half maximum (FWHM = 6.265 px) is annotated to characterize the average speckle grain size. The shaded region represents ±1 standard deviation of the ACF across the dataset. **(D)** Statistical distribution of speckle sizes

(FWHM values) across 500 samples, showing the mean (6.2659 px), standard deviation (0.0816 px), and range (6.08–6.54 px) of the speckle characteristic dimensions. **(E)** Confusion matrix for the baseline 180-state classification. **(F)** Convergence curves for training and validation losses.

Fig. S4.

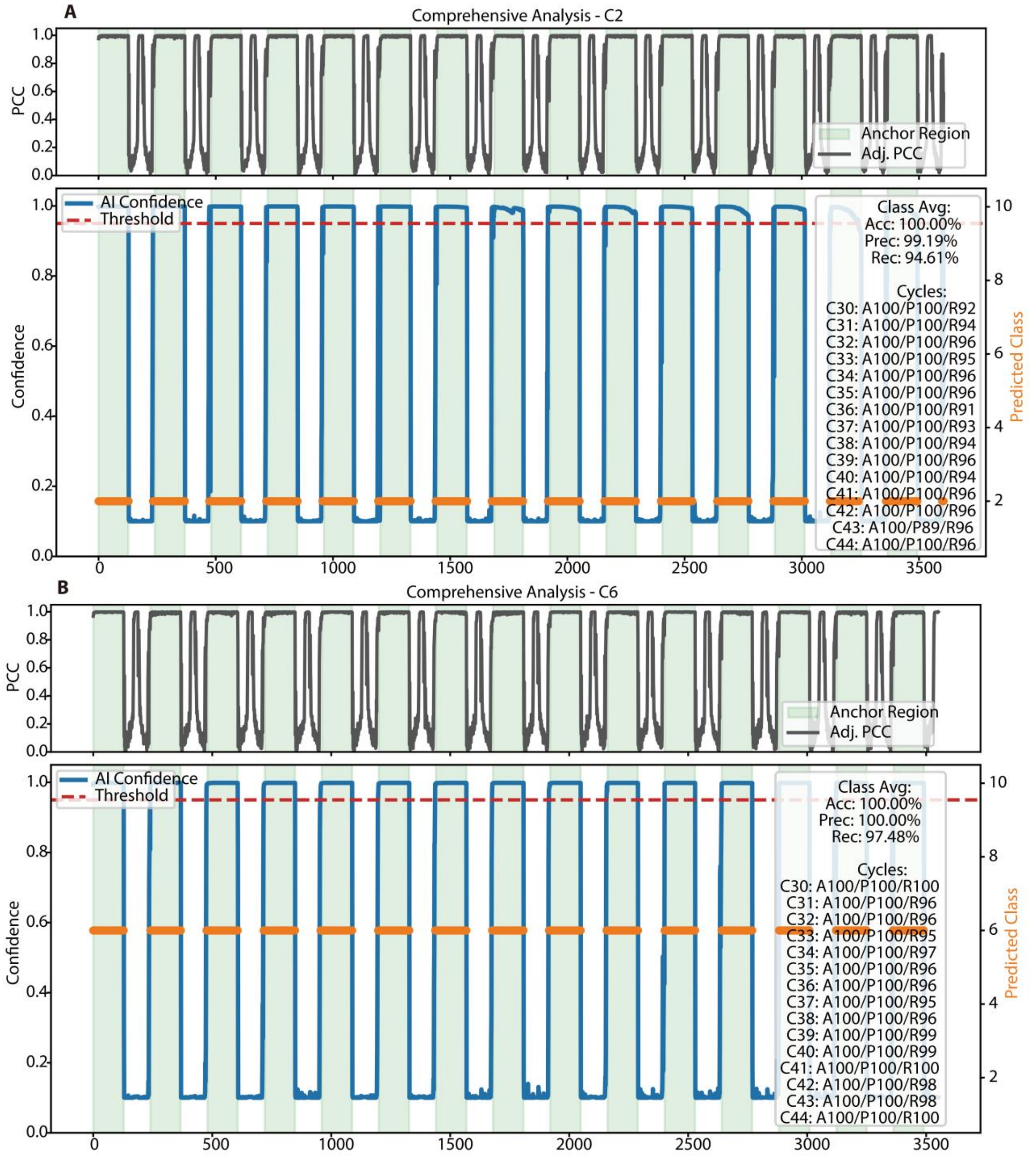

Confidence-gated temporal analyses for additional representative classes. (A) Temporal response characteristics for Class 2. **(B)** Temporal response characteristics for Class 6.

Fig. S5.

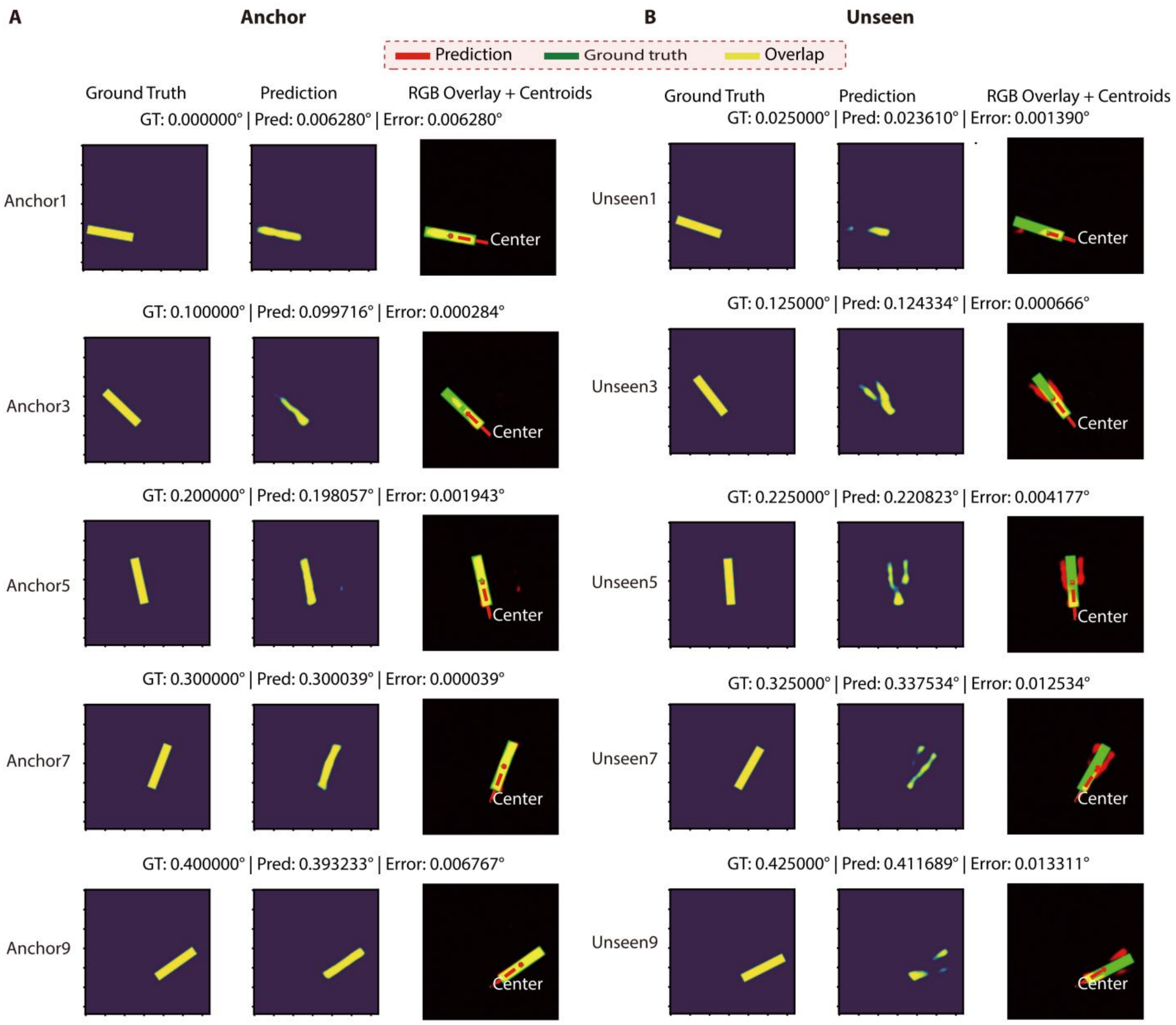

Continuous regression performance using fan-rib geometric encoding. (A) Regression tracking results for the test set of Anchor states, demonstrating the model's fitting accuracy on calibrated data. **(B)** Interpolation results for the test set of Unseen states, validating the model's capability for continuous smooth generalization in uncalibrated intervals.

Fig. S6.

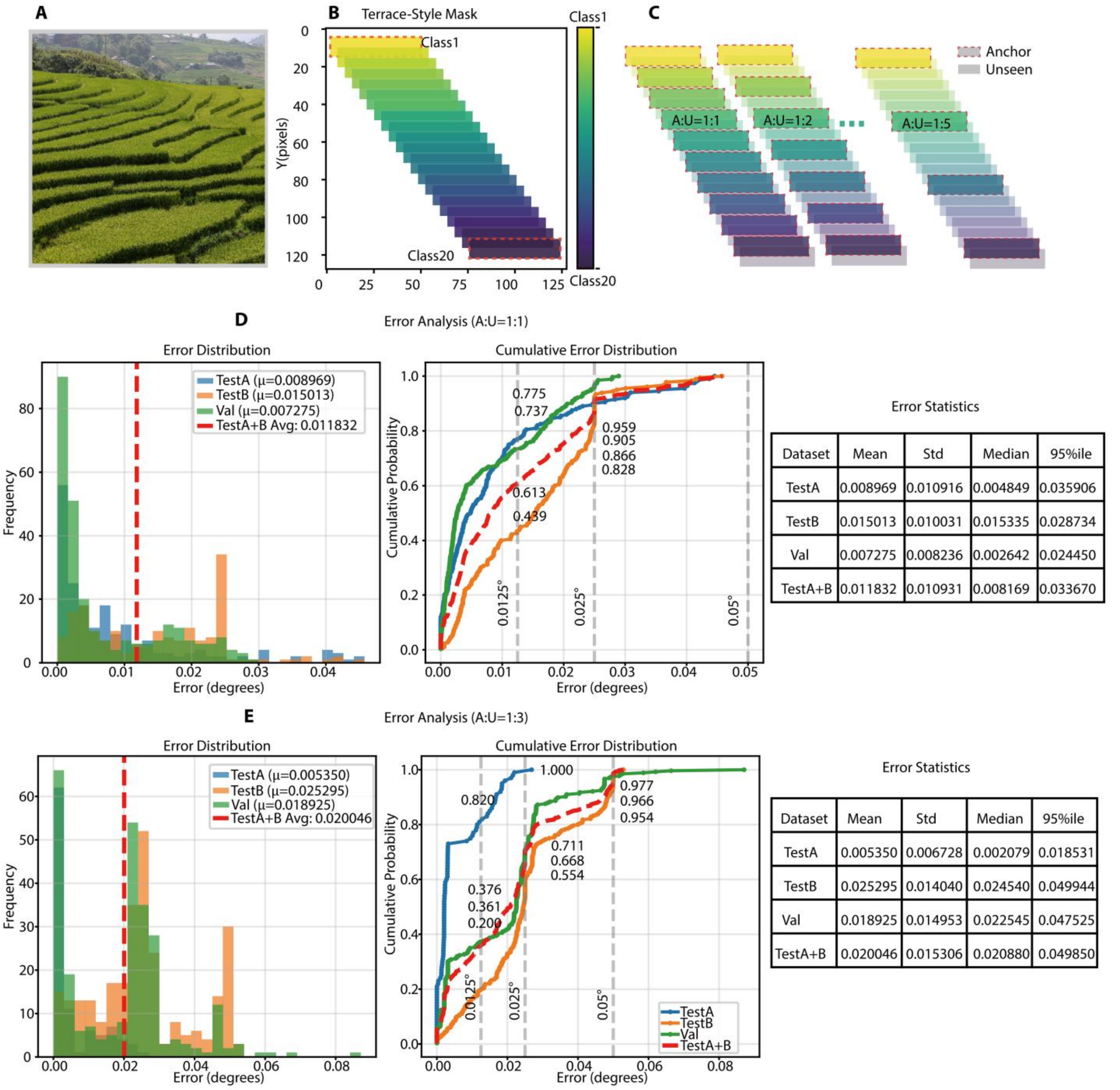

Error Statistics (A:U=1:1)

Dataset	Mean	Std	Median	95%ile
TestA	0.008969	0.010916	0.004849	0.035906
TestB	0.015013	0.010031	0.015335	0.028734
Val	0.007275	0.008236	0.002642	0.024450
TestA+B	0.011832	0.010931	0.008169	0.033670

Error Statistics (A:U=1:3)

Dataset	Mean	Std	Median	95%ile
TestA	0.005350	0.006728	0.002079	0.018531
TestB	0.025295	0.014040	0.024540	0.049944
Val	0.018925	0.014953	0.022545	0.047525
TestA+B	0.020046	0.015306	0.020880	0.049850

Validation of topological universality via alternative "Terrace-type" geometric encoding. (A) Conceptual analogy of terraced fields representing the mapping of continuous variables to spatial positions. **(B)** Definition of the geometric encoding mask for the stepped bending state. **(C)** Data sampling and partitioning strategy under different Anchor-to-Unseen Ratios (AUR). **(D)** Regression error curves and quantitative metrics under full calibration (AUR=1). **(E)** Regression error curves and metrics under sparse calibration (AUR=1:3), highlighting the interpolation limitations of stepped encoding under sparse conditions.

Fig. S7.

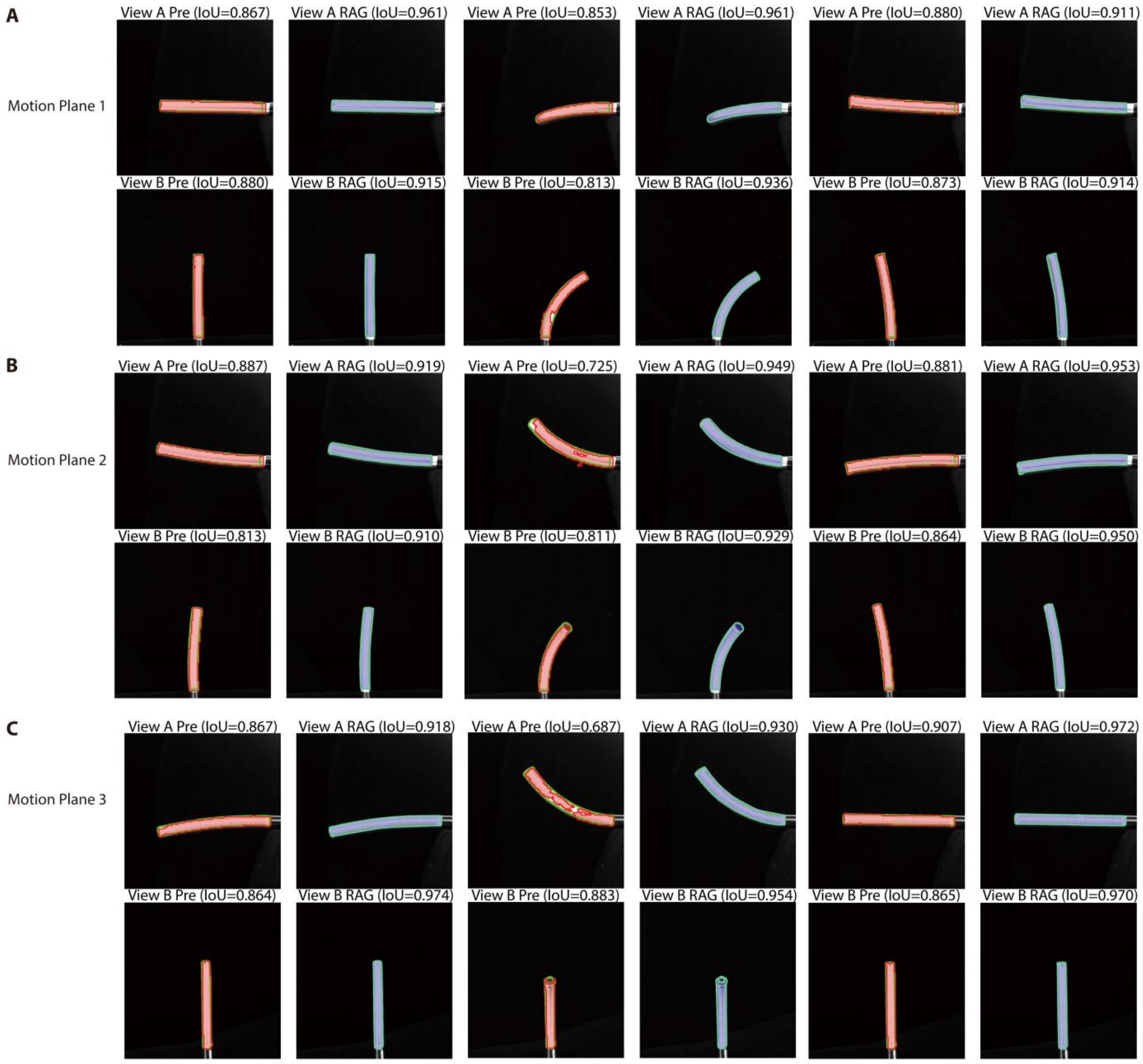

Extended visualization of 3D morphological reconstruction fidelity across distinct motion planes. Detailed reconstruction results for three representative trajectory planes corresponding to the validation experiments.

Fig. S8.

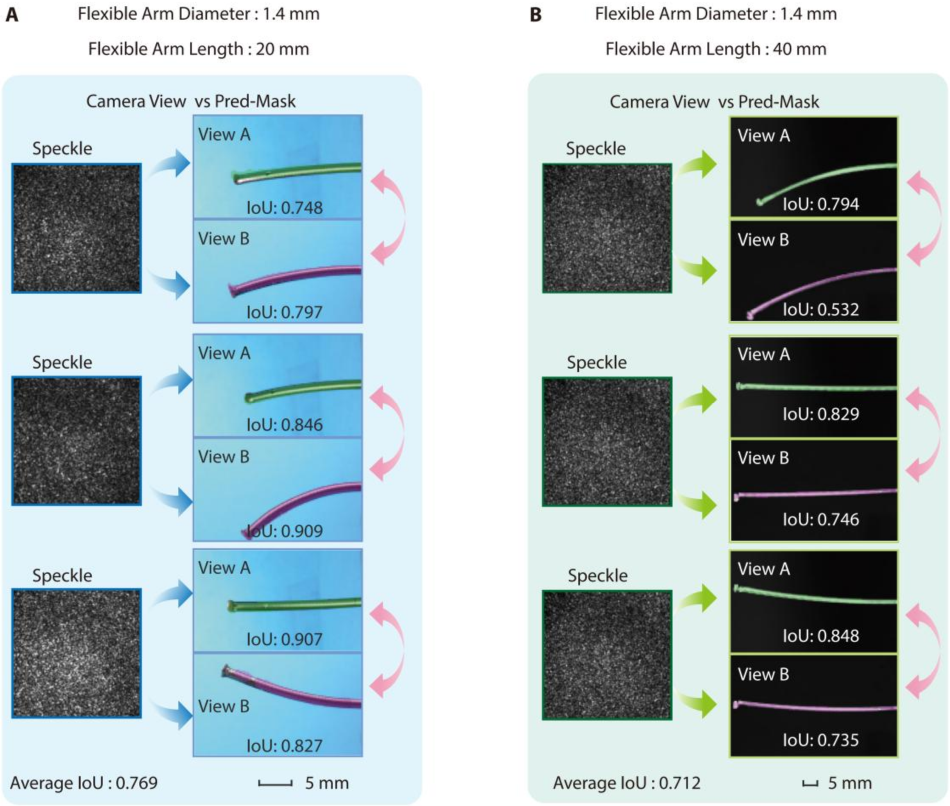

Generalization validation on flexible arms with varying geometric specifications. Representative reconstruction results for flexible arms with distinct aspect ratios (1.4 mm diameter; 20 mm and 40 mm lengths, respectively).

Fig. S9.

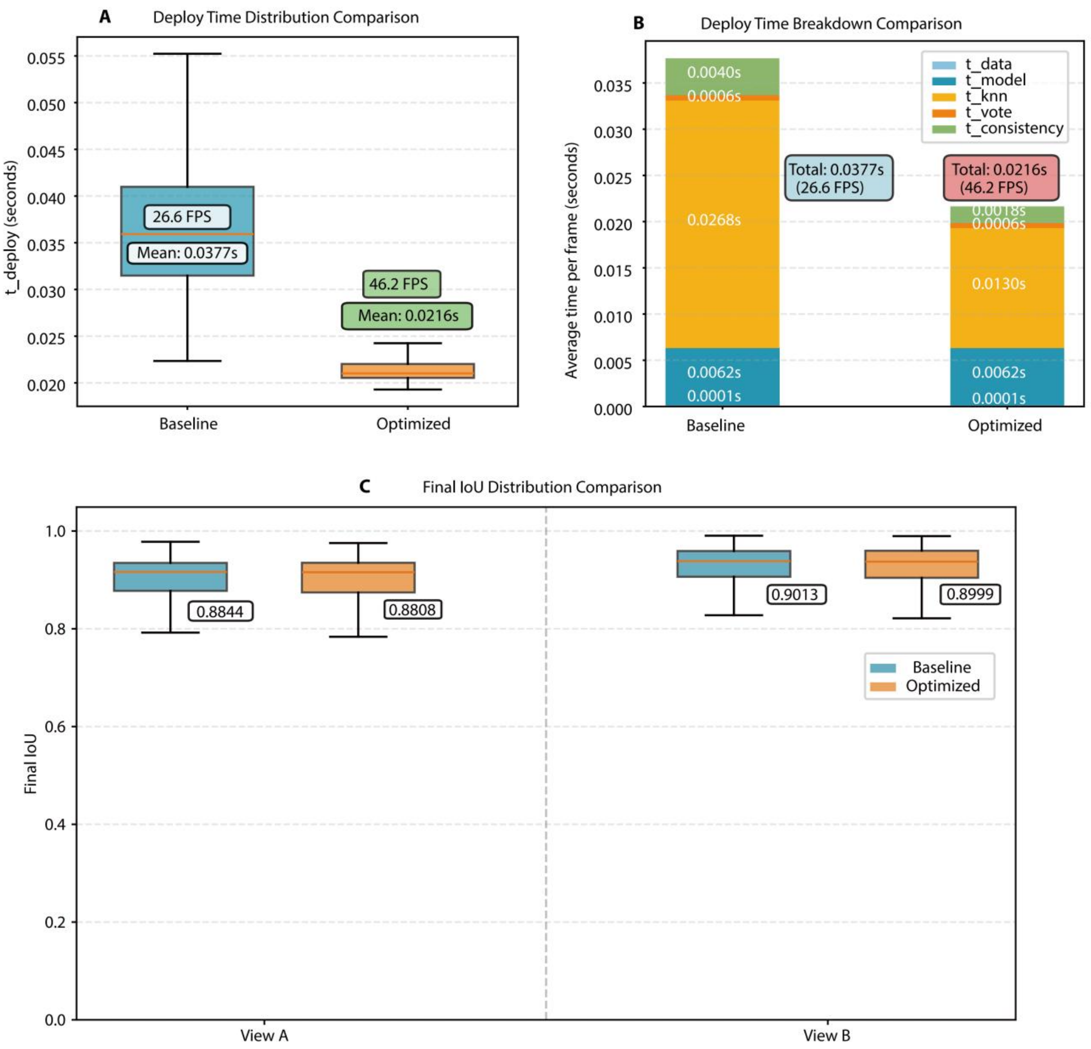

Comparison of deployment latency and segmentation performance between the baseline and optimized versions. **A** Distribution comparison of end-to-end deployment latency between the baseline and optimized versions, with the frame rate (FPS) of the optimized version increased from 26.6 fps to 46.2 fps. **B** Breakdown comparison of end-to-end deployment latency between the baseline and optimized versions. **C** Distribution comparison of the final segmentation IoU of the baseline and optimized versions in two views (View A and View B), where the IoU of the optimized version remains basically unchanged.

Fig. S10.

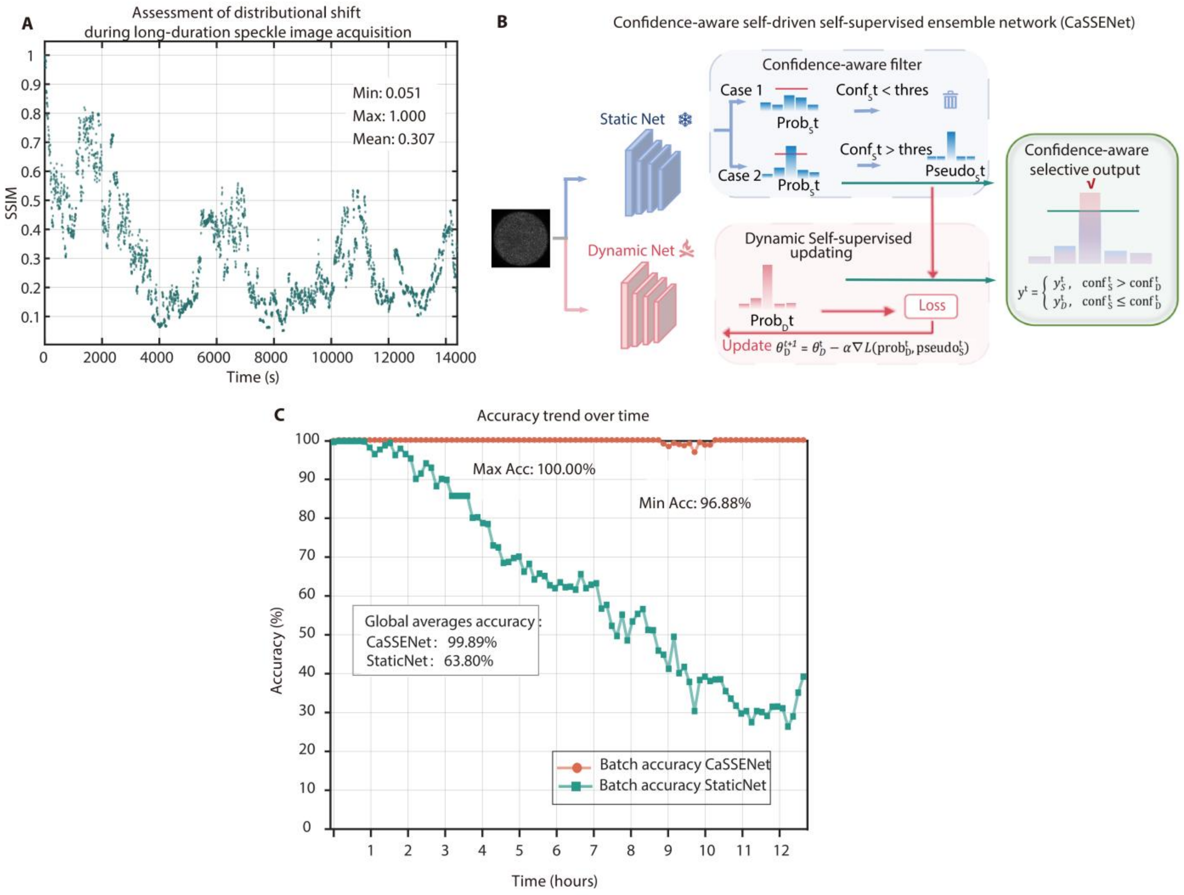

Results of the KL-Divergence-Based Adaptive Learning Strategy. A Assessment of distributional shift during long-duration speckle image acquisition. **B** Framework diagram of the confidence-integrated test-time adaptation algorithm. **C** Accuracy variation curves of the confidence-integrated test-time adaptation algorithm over 12.7 hours of testing in the real-time sensing system. The total number of test data samples is 74,000. Each data point in the figure represents the average value of one batch, with a single batch containing 800 samples.

Fig. S11.

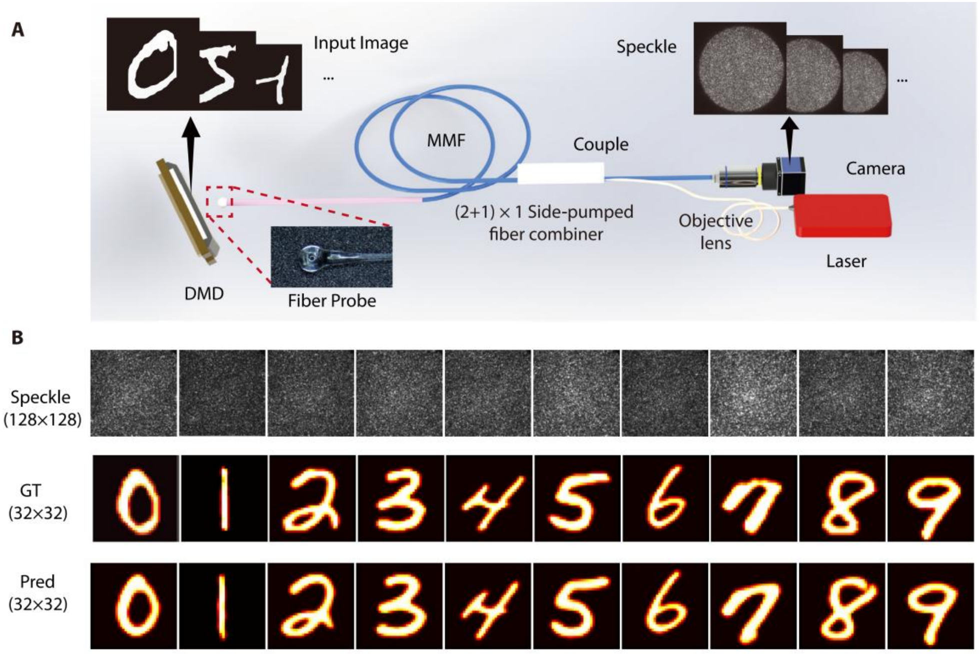

Validation of microscopic imaging capability using the shared MMF architecture. (A) Schematic of the optical path integrated with a Digital Micromirror Device (DMD), utilizing the same MMF hardware as in shape sensing to perform microscopic imaging. **(B)** Imaging reconstruction performance. Comparison among the preprocessed MMF speckle, GT targets, and network-reconstructed images. These results confirm that the sensing fiber retains high-fidelity imaging capabilities, supporting the feasibility of multifunctionality in a single optical channel.

Movie S1.

Dynamic demonstration of MMF output speckle variation with distal end shape changes. This video records in real time the dynamic evolution of the MMF output speckle field in response to continuous morphological changes at the distal end. In the experiment, the deformation curvature of the fiber's distal sensing segment is adjusted stepwise from -90° to 90° (corresponding to the tip angle), with the adjustment step strictly controlled at 1°, fully covering the entire deformation range. This allows for a clear observation of the distinct speckle field patterns corresponding to different spatial shapes, validating the sensitivity of the optical response to geometric deformations.

Movie S2.

Dynamic decoding effect of continuous shape states under the fan-rib encoding method. This video demonstrates the decoding workflow and results for continuous geometric states based on the fan-rib encoding strategy. Taking the fiber's neutral deformation axis as the origin, this method designs a "fan-rib" 2D mask that accurately maps the actual physical curvature. Through a stepwise mapping framework—from speckle patterns to structured masks, and finally to geometric decoding—it achieves high-resolution continuous shape perception. The video intuitively presents the complete decoding pipeline for both anchor states and unseen spatial configurations, including structured mask reconstruction, centroid coordinate calculation, and deformation parameter output, verifying the method's precision in resolving minute shape variations (0.025° resolution).

Movie S3.

Dynamic demonstration of super-resolution mask and skeleton reconstruction via retrieval-augmented generation. This video presents a comprehensive reconstruction test performed on a flexible arm of a soft robot with a deformable length of 30 mm and a diameter of 3 mm across three trajectory planes with distinct motion characteristics. The dynamic reconstruction process highlights the efficacy of the Retrieval-Augmented Generation (RAG) strategy in enhancing morphological fidelity. The video offers a comparison displaying: the raw input speckle fields; the dual-view coarse masks predicted directly by DVS-Net (red outlines), which exhibit visible aliasing artifacts due to resolution limits; and the dual-view refined masks after RAG processing (blue fills). The sequence clearly illustrates how RAG effectively

smoothes boundaries and eliminates pixel-level discontinuities, achieving high alignment with the ground truth (green outlines). Additionally, the corresponding 3D skeleton is synchronized to visualize the spatial posture of the flexible arm.
